# Selective de-repression of germ cell-specific genes in mouse embryonic fibroblasts in a permissive epigenetic environment

**DOI:** 10.1038/srep32932

**Published:** 2016-09-09

**Authors:** Tamotsu Sekinaka, Yohei Hayashi, Toshiaki Noce, Hitoshi Niwa, Yasuhisa Matsui

**Affiliations:** 1 Cell Resource Center for Biomedical Research, Institute of Development, Aging and Cancer (IDAC), Tohoku University, 4-1 Seiryo-machi, Aoba-ku, Sendai, Miyag**i**, 980-8575, Japan; 2 Graduate school of Life Sciences, Tohoku University, 2-1-1 Katahira, Aoba-ku, Sendai, Miyag**i**, 980-8577, Japan; 3 The Japan Agency for Medical Research and Development-Core Research for Evolutional Science and Technology (AMED-CREST), Tokyo 100-0004, Japan; 4 School of Medicine, Keio University, 35 Shinanomachi, Shinjuku-ku, Tokyo 160-8582, Japan; 5 Department of Pluripotent Stem Cell Biology, Institute of Molecular Embryology and Genetics (IMEG), Kumamoto University, 2-2-1 Honjo, Chuo-ku, Kumamoto 860-0811, Japan

## Abstract

Epigenetic modifications play crucial roles on establishment of tissue-specific transcription profiles and cellular characteristics. Direct conversions of fibroblasts into differentiated tissue cells by over-expression of critical transcription factors have been reported, but the epigenetic mechanisms underlying these conversions are still not fully understood. In addition, conversion of somatic cells into germ cells has not yet been achieved. To understand epigenetic mechanisms that underlie germ cell characteristics, we attempted to use defined epigenetic factors to directly convert mouse embryonic fibroblasts (MEFs) into germ cells. Here, we successfully induced germ cell-specific genes by inhibiting repressive epigenetic modifications via RNAi or small-molecule compounds. Under these conditions, some tissue-specific genes and stimulus-inducible genes were also induced. Meanwhile, the treatments did not result in genome-wide transcriptional activation. These results suggested that a permissive epigenetic environment resulted in selective de-repression of stimulus- and differentiation-inducible genes including germ cell-specific genes in MEFs.

The early precursors of germ cells, designated primordial germ cells (PGCs), become established at around embryonic day (E)7.25 in the extraembryonic mesoderm^1^. PGCs then migrate into the indifferent embryonic gonads (genital ridges), and subsequently start to differentiate into sperms or eggs. Developing PGCs express several germ cell-specific genes at specific embryonic developmental stages. For example, nascent PGCs express *Blimp1* (also known as *Prdm1*: PR domain containing 1, with ZNF domain), which is necessary for induction of PGCs^2^; *Stella* (also known as *Dppa3*: developmental pluripotency-associated 3), which is important to embryonic development after fertilization^3^,^4^,^5^ and *Nanos3* (nanos homolog 3), which is necessary for survival of PGCs^6^,^7^. Then, during migration into the genital ridges (E10.5-E13.5), PGCs express *Vasa* (also known as *Ddx4*: DEAD box polypeptide 4), which is important for development of male germ cells^8^,^9^; and PGCs also begin to express meiosis related-genes such as *Dazl* (deleted in azoospermia-like)^10^,^11^ and *Stra8* (stimulated by retinoic acid gene 8) during migration^12^,^13^. Along with those PGC-specific genes, PGC also express pluripotency-associated gene including *Oct4* (also known as *Pou5f1*: POU domain, class5, transcription factor 1), *Sox2* (SRY-box 2), and *Nanog* (Nanog homeobox); these gene products contribute to survival and/or differentiation of PGCs^14^,^15^,^16^,^17^.

During their development, PGC undergo characteristic epigenetic reprogramming. During migration, repressive epigenetic modifications, such as histone H3 Lysine 9 di-methylation (H3K9me2) and DNA methylation, are globally reduced^18^,^19^; simultaneously, histone H3 Lysine 27 tri-methylation (H3K27me3), another repressive histone modification, is elevated^20^. Meanwhile, H3K27 becomes locally hypo-methylated in regulatory regions of germ cell-specific genes prior to their PGC-specific upregulation^21^; these coordinated changes suggest that these epigenetic modifications play important roles in the temporal regulation of germ cell-specific gene expression in PGCs^22^. In addition to those repressive histone modifications, permissive histone modifications also show unique changes in PGCs. For example, H3K4me3 and histone H3 Lysine 9 acethylation (H3K9Ac) are transiently elevated in differentiating PGCs^18^. The importance of some of these epigenetic modifications in embryonic germ cells has been clearly demonstrated. For instance, deficiency of *Meisetz* (also known as *Prdm9*: PR domain containing 9, H3K4 tri-methyltransferase) or of *G9a* (also known as Ehmt2: euchromatic histone lysine N-methyltransferase 2, H3K9 di-methyltransferase) causes abnormal meiosis and infertility^23^,^24^. Although overall physiological meaning of the global epigenetic reprogramming in PGCs is not yet fully understood, this reprogramming might have a role in future establishment of a precise and intricate epigenetic status required for coordinated gene expression after fertilization, and it might be important for PGCs to acquire totipotency^25^.

After undergoing complex differentiation processes that include the above-mentioned epigenetic reprogramming, germ cells acquire totipotency through fertilization and can go on to generate an entire organism, but somatic cells derived from the zygote do not normally have this potential. We reasoned that using defined factors to reconstitute an epigenetic status similar that of germ cells in somatic cells might help us to further understand the cellular characteristics of germ cells at the molecular level.

Reconstitution of pluripotency in somatic cells has been successively achieved with induced pluripotent stem cell (iPSC) by expressing the Yamanaka factors (*Oct4, Sox2, Klf4, c-Myc*) or the Jaenisch factors (*Sall4, Nanog, Esrrb, Lin28*) or via small-molecule compounds (VPA, CHIR99021, ALK5i, Tranylcypromine, Forskolin, DZNep)^26^,^27^,^28^. Studies suggest that the process of the reprogramming is divided into two steps^29^. In the first step, MEFs become dedifferentiated via ectopic expression of Yamanaka factors; consequently, metabolic-associated genes and endogenous Yamanaka factors are upregulated, and somatic genes are downregulated^29^,^30^,^31^. In the second step, the pluripotency-associated gene network is established, and the reprogrammed cells acquire pluripotency^29^,^32^,^33^,^34^.

Direct reprogramming of MEFs into cells of particular somatic tissues has also been reported^35^,^36^,^37^,^38^,^39^. One such reprogramming strategy involves the overexpression of transcription factors that regulate differentiation into particular cell lineages including hepatocytes, neurons, or Sertoli cells. Alternatively, MEFs have been partially reprogrammed by briefly expressing Yanamaka factors (*Oct4, Sox2, Klf4, c-Myc*) or via small-molecule compounds; those partially reprogrammed cells were subsequently induced to become cardiomyocytes or pancreatic cells under the respective culture conditions. Tissue-specific activities of the reprogrammed cells, which were directly converted from MEFs, have been confirmed via *in vitro* functional assays or via *in vivo* transplantation. However, direct reprogramming of MEFs into germ-cell lineages, including PGCs, has not yet been reported.

To recapitulate germ cell characteristics in somatic cells, it is at least necessary to induce pluripotency-associated genes and germ cell-specific genes. To induce pluripotency-associated genes, we simply transfected an expression vector encoding Yamanaka factors (*Oct4, Sox2, Klf4, c-Myc*). To upregulate germ cell-specific genes, we initially examined the effect of *Max* knocked-down (KD) in MEFs, because we previously found that *Max*-KD globally induced germ cell-specific genes in embryonic stem cells (ESCs)^40^. ESCs and PGCs both express pluripotency-associated genes, but germ cell-specific genes are repressed in ESCs, and we suggested that H3K9me2, which can be generated via Max-associating G9a and GLP, is involved in their repression. However, *Max*-KD in MEFs did not show global induction of germ cell-specific genes. Accordingly, we then tested several conditions to alter the epigenetic state of MEFs into that of PGCs.

Here, we describe the preferential induction of stimulus-inducible genes, including germ cell-specific genes; to induce these genes, we used RNAi or small-molecule compounds to inhibit repressive epigenetic modifications such as DNA methylation and H3K27me3. We discuss the hypothesis that this treatment may establish a fundamental cellular status that allows for direct reprogramming of MEFs into multiple cell lineages, including germ cells.

## Results

### Together the Yamanaka factors and *Max*-KD in MEFs resulted in induction of *Vasa* and *Stra8* expression

We attempted to express pluripotency-associated genes and to induce germ cell-specific genes in MEFs to convert MEFs into germ cells. To express pluripotency-associated genes, we transfected an expression vector encoding a tandem set of the Yamanaka factors (*Oct4, c-Myc, Klf4, Sox2*); this construct was designated OCKS^41^. Additionally, we simultaneously used RNAi to knockdown *Max* and thereby induce germ cell-specific genes (Supplementary Fig. S1); again, our previous findings indicate that the *Max* transcription factor globally represses germ-cell specific genes in mouse embryonic stem cells (mESCs), and that *Max* knockdown (*Max*-KD) in mESCs results in upregulation of those genes^40^. Firstly, we confirmed transfection efficiency in MEFs using EGFP expressing plasmids, and found that approximately 20% of the transfected MEFs showed EGFP expression (data not shown). We also examined KD efficiency of *Max* by RNAi, and the expression of *Max* was decreased to 30% of that in control MEFs (Supplementary Fig. S2). After 2 days in culture with the OCKS + *Max*-KD condition, MEFs expressed two typical germ cell-specific genes, *Vasa* and *Stra8* (Supplementary Fig. S3). However, under the condition, expression of the Vasa::RFP reporter was not detected (data not shown), and expression of three other germ cell-specific genes, *Dazl*, *Blimp1*, and *Nanos3*, was not induced (Supplementary Fig. S3). Therefore, we explored additional conditions that might elevate expression of these other germ cell markers.

### Together VPA and ALK5i enhanced Vasa expression

During development of PGCs, histone H3 lysine 9 acethylation (H3K9Ac) is transiently increased^18^, and we reasoned that H3K9Ac elevation might be important to induction of germ cell-specific gene expression. Moreover, transforming growth factor β (TGF-β) represses differentiation of MEF into specific somatic cell lineage^42^. Therefore, we tested the combined effects of a histone deacetylase (HDAC) inhibitor, valproic acid (VPA) and a TGF-β inhibitor (ALK5i) on induction of germ cell-specific genes (Supplementary Fig. S1). We found that VPA and ALK5i together enhanced *Vasa* expression with or without *Max*-KD (Supplementary Fig. S3). However, the VPA-ALK5i treatment (hereafter designated VA5), even with *Max*-KD, could not induce the other germ cell-specific genes (Supplementary Fig. S3) or the Vasa::RFP reporter (data not shown). Thus, we concluded that it was necessary to further explore additional conditions for significant induction of germ cell-specific genes.

### Experimental conditions for induction of germ cell-specific genes

Reportedly, histone H3 lysine 27 tri-methylation (H3K27me3), which is among the repressive histone modifications, is involved in repression of germ-cell specific genes in male germ cells^22^. Additionally, we previously reported that combined knockdown of *Max* and *Atf7ip* (activating transcription factor 7 interacting protein) enhanced Vasa:RFP reporter expression in mESCs^40^. Therefore, we simultaneously knocked down *Max* and *Atf7ip* and separately knocked down *Ezh1* (also known as enhancer of zeste 1 polycomb repressive complex 2 subunit, H3K27 tri-methyltransferase) and *Ezh2* (also known as enhancer of zeste 2 polycomb repressive complex 2 subunit, H3K27 tri-methyltransferase) with or without *Max*-KD (Supplementary Fig. S1, S4a). The simultaneous knockdown of *Max* and *Atf7ip* resulted in higher *Vasa* expression than did the tripled knockdown of *Max*, *Ezh1*, and *Ezh2*. Next, we combined simultaneous knockdown of *Max* and *Atf7ip* with VA5 treatment that showed enhancement of *Vasa* (Supplementary Fig. S3). Consequently, this condition resulted in highest level of *Vasa* induction that we observed (Supplementary Fig. S4b), although the expression levels of *Vasa* in this condition was low compared to that in E13.5 ♂ PGCs (Supplementary Fig. S10a).

We also tested molecules that inhibit the repressive histone modifications (Supplementary Fig. S1). Notably, a combination of three inhibitors—tranylcypromine, which inhibits H3K4 demethylation; BIX-01294, which inhibits H3K9 methylation; and 3′-deazaneplanocin A (DZNep), which inhibits H3K27 methylation (hereafter designated chem)—together with VPA, ALK5i, and the OCKS construct, also induced *Vasa* expression (Supplementary Fig. S4c), although the expression levels of *Vasa* in this condition was low compared to that in E13.5 ♂ PGCs (Supplementary Fig. S10a).

**Figure 1:**
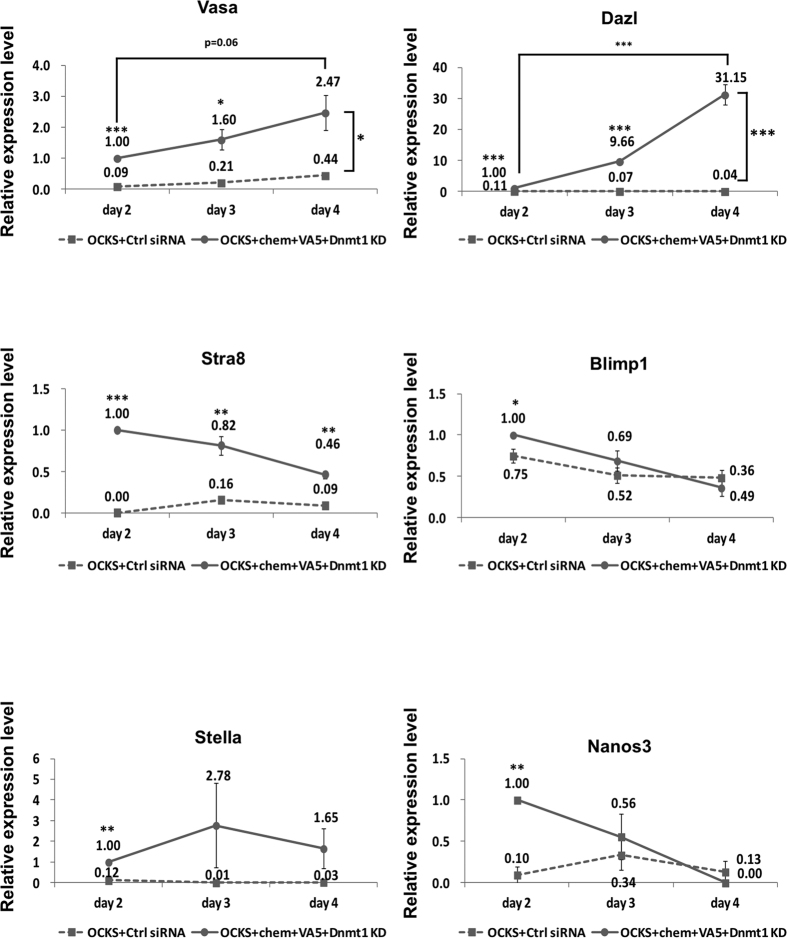
Changes of germ cell-specific gene expression in MEFs in OCKS + chem + VA5 + *Dnmt1*-KD condition. The expression of germ cell-specific genes was quantified by real-time PCR in MEFs after transfection of the expression vector encoding the Yamanaka factors (*Oct4, c-Myc, Klf4, Sox2*: OCKS) with or without addition of tranylcipromine, BIX-01294, DZNep (chem), VPA, ALK5i (VA5), and *Dnmt1* Knocked-Down (KD) (OCKS + chem + VA5 + *Dnmt1*-KD, or OCKS + Ctrl siRNA) after 2, 3, 4 days in culture. The expression level of each gene in MEFs with OCKS + chem + VA5 + *Dnmt1*-KD was set as 1.0. Error bars: S.E. of three biological replicates, *p < 0.05, **p < 0.01, ***p < 0.001 (Student’s t-test).

Reportedly, inhibition of DNA methylation in mESCs results in induction of germ-cell-specific genes including *Dazl* and *Stella*^21^. Therefore, we tested the OCKS + *Max*-KD + *Atf7ip*-KD + VA5 combination or the OCKS + chem + VA5 combination with either of two inhibitors of DNA methylation, *Dnmt1*-KD or 5-Aza-cytidine (Aza) (Supplementary Fig. S1); the OCKS + chem + VA5 + *Dnmt1*-KD combination significantly induced *Dazl*, *Stra8*, *Blimp1*, and *Stella* in addition to *Vasa* after 2 days in culture (Supplementary Fig. S5a). Moreover, *Vasa* and *Dazl* expression was further elevated in a culture period-dependent manner until 4 days (Fig. 1). However, the expression of Vasa tended to decrease after 6 in culture (Supplementary Fig. S6), and the expression from the Vasa::RFP reporter was not detectable after 2 weeks in culture (data not shown). Meanwhile, the OCKS + chem + VA5 + *Dnmt1*-KD combination did not induce somatic genes such as *Hoxa1* and *Hoxb1* (Supplementary Fig. 5b); this finding suggested that induction of germ cell-specific genes by OCKS + chem + VA5 + *Dnmt1*-KD was not due to non-specific transcription activation.

### The Yamanaka factors were not necessary for induction of *Vasa* and *Dazl* expression

**Figure 2:**
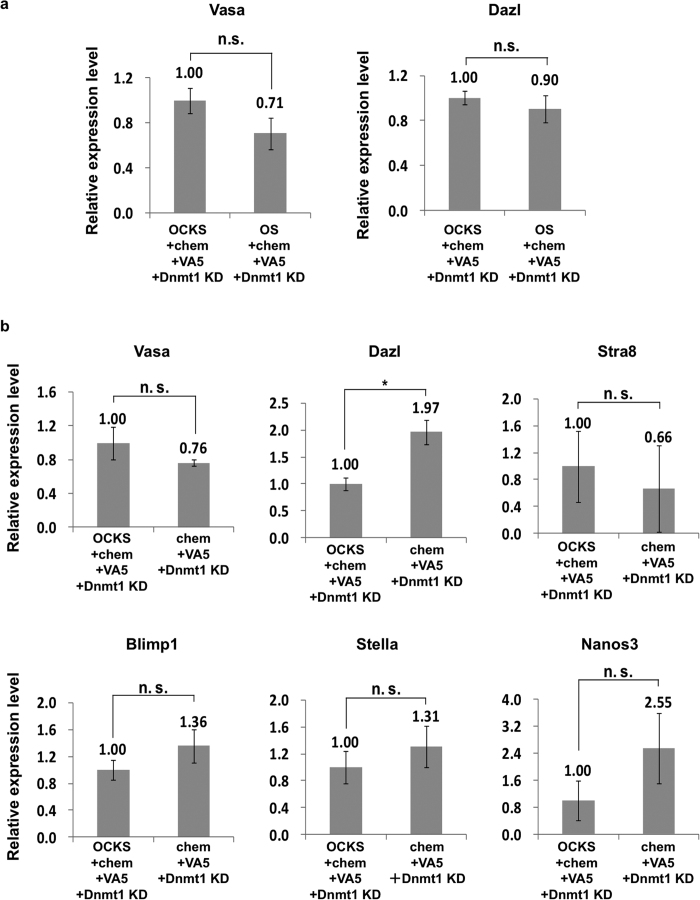
Yamanaka factors were not necessary for induction of germ cell-specific genes. **(a)** *Vasa* and *Dazl* expression in the presence of OCKS or OS with chem + VA5 + *Dnmt1*-KD after 4 days in culture. **(b)** The expression of germ cell-specific genes with or without OCKS in chem + VA5 + *Dnmt1*-KD condition after 2 days in culture. The expression of germ cell-specific genes was quantified by real-time PCR. The expression level of each gene in MEFs with OCKS + chem + VA5 + *Dnmt1*-KD was set as 1.0. Error bars: S.E. of three biological replicates, *p < 0.05, n.s.: not significantly different (Student’s t-test).

Because *Klf4* and *c-Myc* expression were not detectable in PGCs as reported previously^43^, they may not be necessary for germ cell-specific genes induction. Accordingly, we compared germ cell-specific gene expression with or without *Klf4* and *c-Myc* in the presence of chem + VA5 + *Dnmt1*-KD. *Vasa* and *Dazl* expression did not differ significantly between treatment groups (Fig. 2a). Additionally, induction of *Vasa*, *Dazl*, and other germ cell-specific genes was not significantly changed even in the absence of all Yamanaka factors (Fig. 2b). These data suggested that germ-cell specific gene expression in MEFs was regulated by epigenetic modifications, but was independent of the reprogramming factors.

### Dazl protein was significantly induced in the treated MEFs

**Figure 3:**
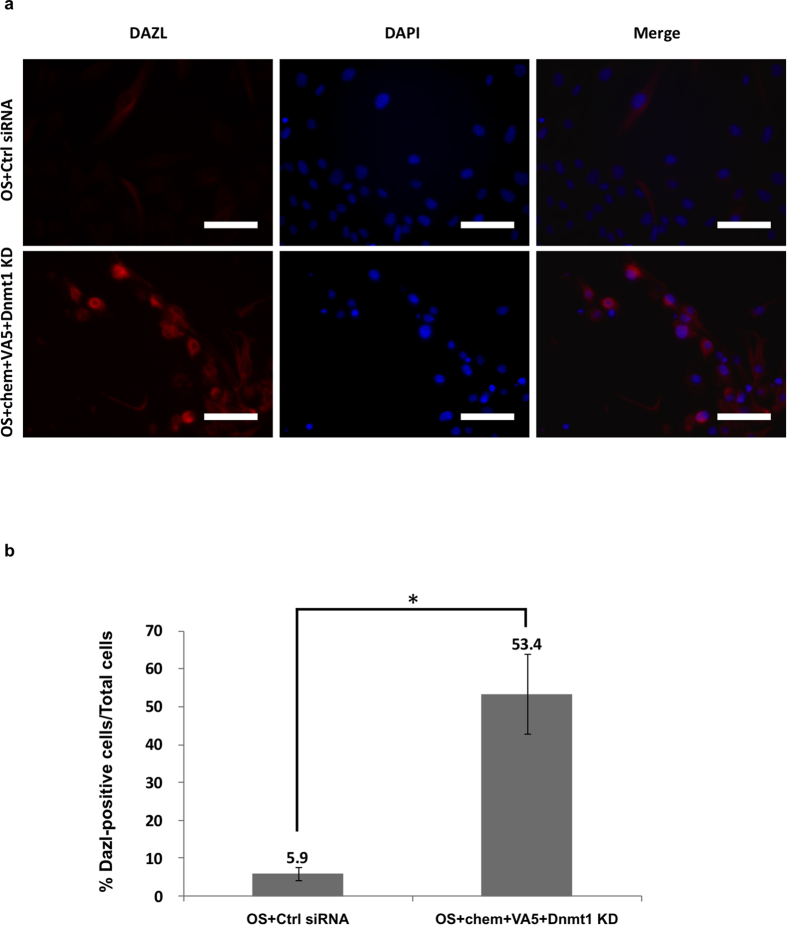
Dazl protein was significantly increased in MEFs following OS + chem + VA5 + *Dnmt1*-KD treatment. **(a)** Immuno-fluorescence staining of MEFs after 4 days in culture with or without chem + VA5 + *Dnmt1*-KD in the presence of OS using anti-Dazl antibody. The shown data are representative of three independent experiments. Red: anti-Dazl, Blue: DAPI, scale bar: 200 μm. **(b)** The ratios of Dazl-positive cells in DAPI-positive cells. Numbers of Dazl- or DAPI-positive cells was estimated by image J. Error bars: S. E. of three biological replicates, *p < 0.05.

To estimate the proportion of MEFs that expressed germ-cell specific genes following induction via OCKS or OS  + chem + VA5 + *Dnmt1*-KD treatments, we used anti-Dazl antibody to immunostain cultured cells. Notably, Dazl protein was detected in about 50% of of the treated MEFs (Fig. 3; Supplementary Fig. S7). Meanwhile only about 4–7% of the intact MEFs as well as of OCKS + Ctrl siRNA treated MEFs showed the expression of Dazl protein (Fig. 3; Supplementary Fig. S7, data not shown). These findings suggested that upregulation of germ cell-specific genes may generally occur in MEFs after OCKS or OS  + chem + VA5 + *Dnmt1*-KD treatments.

### Genome-wide analysis of gene expression in treated MEFs

**Figure 4:**
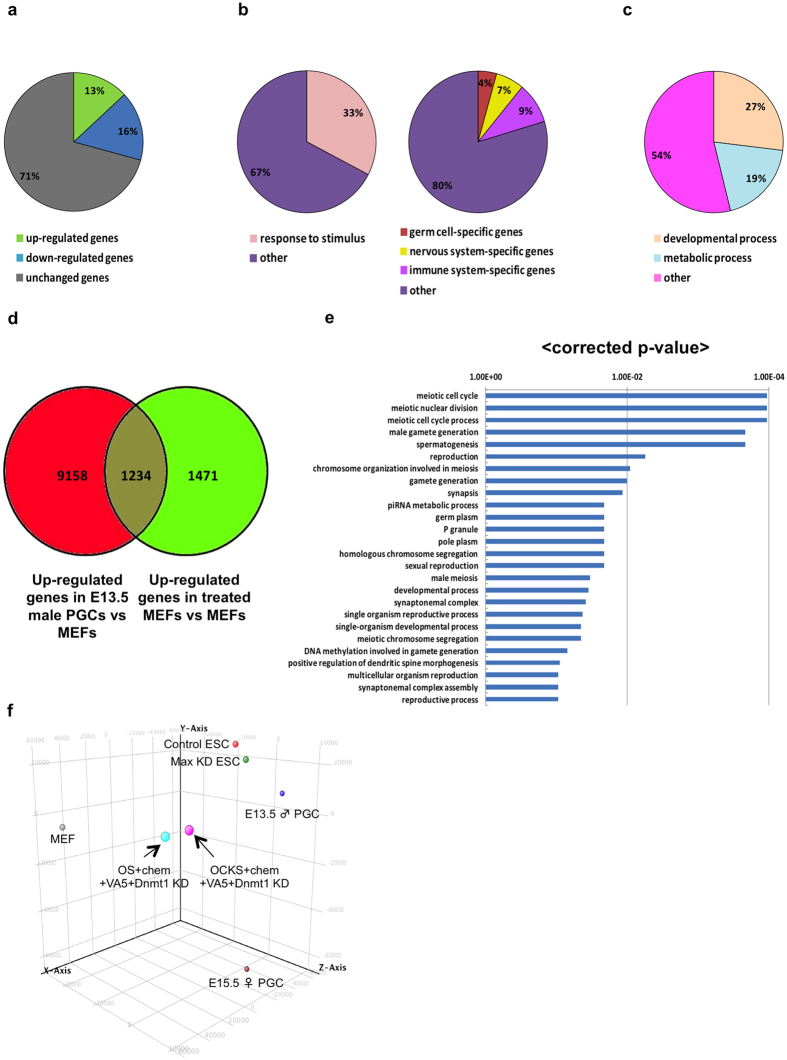
Transcriptome analysis of MEFs in OS + chem + VA5 + *Dnmt1*-KD condition. (**a**) The ratios of number of upregulated or downregulated genes in MEFs 4 days after OS + chem + VA5 + *Dnmt1*-KD treatment compared with in control MEFs. Genes whose expression was changed at least two-fold (p < 0.001) were analyzed. **(b)** The ratios of upregulated genes annotated with particular GO terms. **(c)** The ratios of downregulated genes annotated with particular GO terms. **(d)** Relationship of upregulated genes in E13.5 male PGCs and in the treated MEFs compared with control MEFs. **(e)** GO analysis in commonly upregulated genes in E13.5 male PGCs and in the treated MEFs compared with control MEFs. The GO terms with corrected p-value under 0.05 are shown. **(f)** Principal Component Analysis (PCA) of control MEFs (MEF), MEFs with OS or OCKS + chem + VA5 + *Dnmt1*-KD treatments after 4 days in culture, E13.5 male (♂) PGCs, E15.5 female (♀) PGCs, control ESCs, and Max KD ESCs. X-Axis: Component 1 (46.49%), Y-Axis: Component 2 (26.37%), Z-Axis: Component 3 (11.12%). The array data obtained from three biological replicates.

To understand genome-wide changes in gene expression following OCKS or OS  + chem + VA5 + *Dnmt1*-KD treatments, we compared the transcriptome of treated MEFs with that of control MEFs. The results indicated that 13% and 16% of genes were upregulated or downregulated, respectively, following OS  + chem + VA5 + *Dnmt1*-KD treatments (Fig. 4a). Similar results were also obtained following treatment with the OCKS construct that encoded four reprogramming genes (Supplementary Fig. S8a). The upregulated genes in the treated MEFs included not only germ cell-specific genes, but also tissue-specific genes such as nervous system- and immune system-specific genes, as well as a number of stimulus-inducible genes (Fig. 4b; Supplementary Fig. S8b). In the case of OCKS-treated cells, metabolic process-related genes were also upregulated (Supplementary Fig. S8b). The downregulated genes in the treated MEFs included genes related to developmental processes or to metabolic processes (Fig. 4c; Supplementary Fig. S8c).

To understand relationship between PGCs and the treated MEFs, we compared the transcriptome of male PGCs from E13.5 mouse embryos and that of the treated MEFs to the transcriptome of control MEFs. We found that 1234 genes were upregulated in both PGCs and OS-treated MEFs, and 1427 genes were upregulated in both PGCs and OCKS-treated MEFs; notably, meiosis-related GO terms and genes including Tex19.1, Dazl, Sycp1 and Sycp3 were highly enriched in both sets of up-regulated genes (Fig. 4e; Supplementary Fig. S8e; Supplementary Fig. S9; Supplementary Table S3; Supplementary Table S4). The results indicated that germ cell-specific genes were selectively upregulated in MEFs by either treatment. Additionally, we performed principal component analysis (PCA) of control MEFs, MEFs with OS or OCKS + chem + VA5 + *Dnmt1*-KD, E13.5 male (♂) PGCs, E15.5 female (♀) PGCs, control ESCs, and *Max*-KD ESCs (Fig. 4f). We found that OS- and OCKS-treated MEFs were closely correlated with each other, supporting the idea that *Klf4* and *c-Myc* did not significantly affect the transcriptome of MEFs cultured with OS + chem + VA5 + *Dnmt1*-KD. Meanwhile, the transcriptome of the treated MEFs was positioned midway between control MEFs and PGCs; this finding indicated that the OS or OCKS + chem + VA5 + *Dnmt1*-KD conditions substantially altered transcriptome of MEFs towards that of PGCs, and that establishment of a germ cell-like transcriptome in MEFs would require additional manipulation.

### Meiosis-related genes were repressed by DNA methylation and histone H3 lysine 27 tri-methylation in MEFs

**Figure 5:**
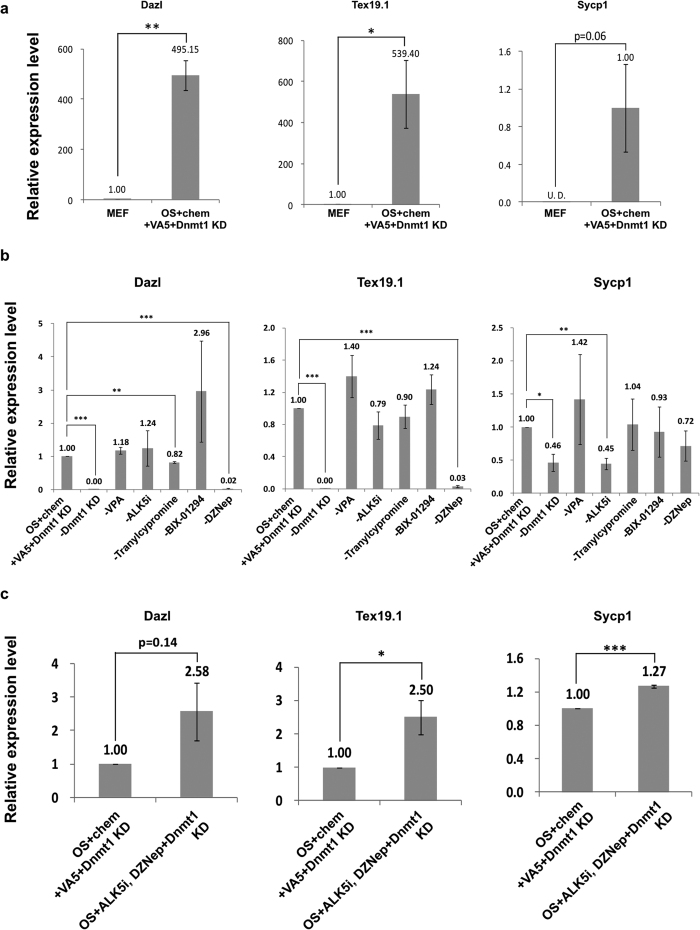
Inhibition of DNA methylation, TGF-β, and H3K27me3 was essential for *Dazl*, *Tex19.1*, and *Sycp1* induction. **(a)** The expression of *Dazl*, *Tex19.1* and *Sycp1* in MEFs after 4 days in culture following OS + chem + VA5 + *Dnmt1*-KD treatment and in control MEFs. The expression in control MEFs for *Dazl* and *Tex19.1,* or in MEFs in OS + chem + VA5 + *Dnmt1*-KD condition for *Sycp1* was set as 1.0. **(b)** The effects of removal of each inhibitor from OS + chem + VA5 + *Dnmt1*-KD condition on *Dazl*, *Tex19.1*, and *Sycp1* expression after 4 days in culture. The expression in MEFs following OS + chem + VA5 + *Dnmt1*-KD treatment was set as 1.0. **(c)** The expression of *Dazl*, *Tex19.1*, and *Sycp1* in MEFs after 4 days in culture under OS + chem + VA5 + *Dnmt1*-KD or OS + ALK5i + DZNep + *Dnmt1*-KD conditions. The expression in MEFs in the OS + chem + VA5 + *Dnmt1*-KD condition was set as 1.0. The expression of *Dazl, Tex19.1* and *Sycp1* was quantified by real-time PCR. Error bars: S.E. of three biological replicates, *p < 0.05, **p < 0.01, ***p < 0.001, n.s.: not significantly different (Student’s t-test).

Finally, we investigated the epigenetic regulatory mechanisms responsible for upregulation of meiosis-related genes in MEFs subjected to the OS + chem + VA5 + *Dnmt1*-KD treatment. We focused on *Dazl*, *Tex19.1*, and *Sycp1* because they were highly upregulated in the treated MEFs (Fig. 5a; Supplementary Fig. S9; Supplementary Table S3; Supplementary Table S4). Meanwhile, the expression of those genes in the treated MEFs was lower than that in E13.5 PGCs (Supplementary Fig. S10b), indicating that OS  + chem + VA5 + *Dnmt1*-KD treatment is still not enough to fully up-regulate the germ cell-specific gene expression. We assessed which treatments were essential for induction of *Dazl*, *Tex19.1*, and *Sycp1* and found that inhibition of DNA methylation, of H3K27me3, and of TGF-β critically affected induction of these genes (Fig. 5b). In addition, only these three inhibitors in combination with OS (OS + ALK5i + DZNep + *Dnmt1*-KD) were able to induce levels of *Dazl*, *Tex19.1*, and *Sycp1* expression comparable to the OS  + chem + VA5 + *Dnmt1*-KD treatment (Fig. 5c). Similar results were also obtained in ALK5i + DZNep + *Dnmt1*-KD condition without OS (Supplementary Fig. S11), again suggesting that OS expression is not critical for induction of those germ cell-specific genes.

**Figure 6:**
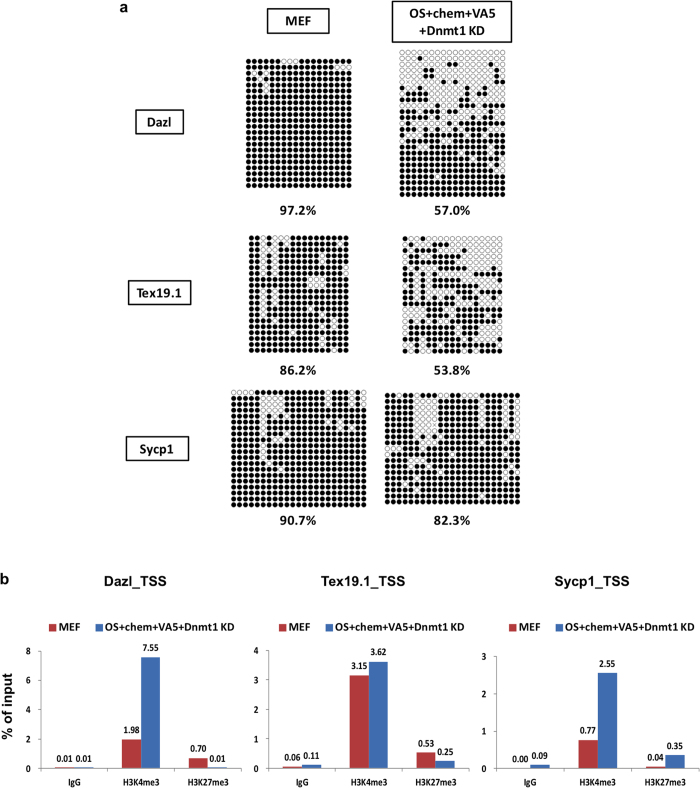
Changes in epigenetic modifications in MEFs following OS + chem + VA5 + *Dnmt1*-KD treatment. **(a)** The DNA methylation status in the *Dazl*, *Tex19.1*, and *Sycp1* promoter regions in MEFs 4 days after the OS + chem + VA5 + *Dnmt1*-KD treatment or in control MEFs. DNA methylation status was determined by bisulphite sequencing. The filled and open circles indicate methylated- and un-methylated CpGs, respectively. The data shown were combined from two independent experiments. The percentage of methylated-CpGs is shown. **(b)** ChIP analysis of the promoter regions of *Dazl, Tex19.1*, and *Sycp1* for H3K4me3 and H3K27me3 in MEFs 4 days after the OS + chem + VA5 + *Dnmt1*-KD treatment or in control MEFs. ChIP using normal IgG was used as negative control. Levels of H3K4me3 or H3K27me3 were determined by real-time PCR, and the percentages of values relative to those for input chromatin are shown. Representative data from two independent experiments are shown. See also Supplementary Fig. S9.

Next, to determine whether any treatment changed the DNA methylation or H3K27me3 levels in the promoter regions of *Dazl*, *Tex19.1*, or *Sycp1*, we performed bisulphite sequencing and chromatin immunoprecipitation (ChIP) assays. The promoter region of each gene became hypo-methylated, though the change in *Sycp1* was relatively small (Fig. 6a). At the *Dazl* and *Tex19.1* promoters in MEFs, H3K27me3 levels were also reduced following OS + chem + VA5 + *Dnmt1*-KD treatments. (Fig. 6b; supplementary Fig. S12). Moreover, both H3K4me3 and H3K27me3 were enriched in the promoter regions of *Dazl* and *Tex19.1* (Fig. 6b; supplementary Fig. S12); therefore, both of those genes can maintain bivalent histone modifications. Interestingly, H3K4me3 and H3K27me3 levels at *Scyp1* were higher in the treated MEFs than in control MEFs (Fig. 6b; Supplementary Fig. S12).

These results suggested that the expression of those three genes was repressed by DNA methylation. In addition, H3K27me3 was also involved in repression of *Dazl* and *Tex19.1*. Meanwhile, *Sycp1* may be regulated by different mechanisms than those regulating *Dazl* and *Tex19.1*.

## Discussion

Although our previous study reported that *Max*-KD ESCs show global induction of germ-cell specific genes^40^, *Max*-KD MEFs did not (Supplementary Fig. S3). This difference may be partly due to differences between ESCs and MEFs with regard to epigenetic status; for example, the genome of MEFs is relatively hyper-methylated compared with that of ESCs^9^,^19^. Therefore, we suspected that a more permissive epigenetic environment, one resembling that of ESCs or PGCs, was necessary for global induction of germ cell-specific genes in MEFs.

During the exploration for conditions for generating epigenetic environments permissive for induction of germ cell-specific genes, we found that *Vasa* was more readily induced under different conditions than other germ cell-specific genes (Supplementary Fig. S3); this finding suggested that *Vasa* differed from other germ cell-specific genes in its sensitivity to alter epigenetic environments. Ultimately, we found that the simultaneous inhibition of repressive epigenetic modifications, including inhibition of DNA methylation and of some repressive histone modifications, induced several germ cell-specific genes (Supplementary Fig. S5). In particular, simultaneous inhibition of DNA methylation, H3K27me3, and TGF-β signaling was important for *Dazl*, *Tex19.1*, and *Sycp1* induction (Fig. 5; Supplementary Fig. S11).

In about 50% of the treated MEFs, Dazl protein was upregulated (Fig. 3, Supplementary Fig. S7), suggesting that at least some germ cell-specific genes were upregulated in half of MEFs following treatment with OCKS or OS + chem + VA5 + *Dnmt1*-KD. Meanwhile, other germ cell-specific gene products, Vasa and Sycp3 protein were not detectable in MEFs after either treatment (Supplementary Fig. S13; Supplementary Fig. S14). In addition, expression of the germ cell-specific reporters Vasa::RFP and Oct4ΔPE-GFP was also not detected. It indicates that the expression of those reporters was under sensitivity of detection by a fluorescence microscope, while RT-qPCR has higher sensitivity to detect mRNA of the endogenous Vasa. These results suggested that both the expression levels of germ cell-specific genes and the number of upregulated germ cell-specific genes were not high enough to drive germ cell differentiation. In fact, levels of germ cell-specific gene expression in the treated MEF was low compared to that in E13.5 PGCs (Supplementary Fig. S10) and meiosis-like phenotypes such as formation of synaptonemal complexes were not observed under this condition (Supplementary Fig. S14).

Transcriptome analysis revealed that OCKS or OS + chem + VA5 + *Dnmt1*-KD treatment resulted in global changes in MEF transcription such that it became more like PGC transcription (Fig. 4f), and germ cell-specific genes were selectively induced in the treated MEFs (Fig. 4b,d,e, Supplementary Fig. S8b,d,e), though the overall transcriptome profile of the treated MEFs was still different from that of PGCs (Fig. 4f). Meanwhile, the PCA showed that the transcriptomes of MEFs in OCKS or OS + chem + VA5 + *Dnmt1*-KD conditions were not close to ESCs, which suggested that the conditions did not result in the enhancement of reprogramming into pluripotential stem cells. Consistent to this idea, iPSC colony formation was severely inhibited in OCKS + chem + VA5 + Dnmt1 KD condition (Supplementary Fig. S15).

The induced permissive epigenetic modifications in this study resulted in up-regulation of only a part of genes expression, but not in global de-repression of transcription (Fig. 4, Supplementary Fig. S8); only some genes such as tissue-specific genes including germ cell-specific genes, nervous system-specific genes and immune system-specific genes were selectively upregulated, but about two third of genes including somatic genes such as Hoxa1 and Hoxb1 were unchanged (Fig. 4, Supplementary Fig. S8, Supplementary Fig. S5b). This finding suggested that the changes in epigenetic modifications selectively influenced transcriptional activity of some genes in the treated MEFs, and similar epigenetic transcriptional machineries may commonly repress the expression of germ cell-, nervous system- and immune system-specific genes. Because tissue-specific genes are generally induced in response to extra-cellular signaling stimuli, genes induced by several different extra-cellular stimuli might be relatively sensitive to the permissive epigenetic environments such as hypo-methylation of DNA and of H3K27 in MEFs. Therefore, we tested the effects of BMP4, BMP8b, SCF, and EGF, which are each important for PGC-like cell induction from pluripotent cells^44^. However, only *Blimp1* was marginally upregulated in MEFs following treatment with OS + chem + VA5 + *Dnmt1*-KD and those growth factors (Supplementary Fig. S16). Therefore, in addition to the manipulation of epigenetic status, expression of critical transcription factors might be needed to further enhance transcription of a group of genes specific to germ cells to drive the differentiation of MEFs into germ cells.

Transcriptome analysis also revealed that metabolic-related genes were upregulated under OCKS + chem + VA5 + *Dnmt1*-KD conditions, but not without *Klf4* and *c-Myc* (Fig. 4, Supplementary Fig. S8). This difference of upregulation of metabolic-related genes may be partly due to *c-Myc* over-expression, because a previous study indicated that *c-Myc* caused upregulation of metabolic- and cell cycle-related genes in MEFs^29^. Except for the upregulation of metabolic-related genes, the transcriptomes of OS + chem + VA5 + *Dnmt1*-KD-treated MEFs with or without *Klf4* and *c-My*c were similarly changed relative to control MEFs (Fig. 4f).

The expression of germ-cell specific genes was not affected by OCKS (Fig. 2), which indicated that the expression of pluripotency-associated genes and the consequent acquisition of pluripotency was not a prerequisite for germ cell-specific gene induction.

In this study, we examined three meiosis-related genes, *Dazl*, *Tex19.1*, and *Sycp1*, to assess changes in epigenetic status after treatment of MEFs. We found that the transcription start site (TSS) of those genes became hypo-methyalted, although the TSS of *Sycp1* was less affected than those of the other two (Fig. 6). In addition, *Dazl* and *Tex19.1* showed the bivalent histone modification, i.e., concomitant modifications of H3K4me3 and of H3K27me3 in MEFs (Fig. 6b, Supplementary Fig. S12). Under OS or OCKS + chem + VA5 + *Dnmt1*-KD conditions, H3K27me3 levels were reproducibly reduced at *Dazl* and *Tex19.1*. The results suggested that those three genes were repressed by DNA methylation, and *Dazl* and *Tex19.1* were also poised by the bivalent histone modifications in MEFs. This hypothesis is consistent with the finding that removal of *Dnmt1*-KD or DZNep severely affects induction of *Dazl* and *Tex19.1* (Fig. 5b). Meanwhile increased H3K4me3 may be important for induction of *Sycp1* (Fig. 6b, Supplementary Fig. S12). In addition to those direct epigenetic changes, the possible indirect influence of TGF-β signaling, which is known to inhibit reprogramming of MEFs into iPS cells^42^, may also repress some germ cell-specific genes such as *Sycp1*, because removal of ALK5i, a potent inhibitor of TGF-β signal, significantly impaired induction of *Sycp1* (Fig. 5b).

In conclusion, different epigenetic mechanisms may be involved in repression of germ cell-specific genes in MEFs. Further detailed examination of those epigenetic mechanisms that are responsible for germ cell-specific gene repression may lead to a greater understanding of fundamental differences between somatic cells and of germ cells.

## Methods

### Cell culture

Mouse embryonic fibroblasts (MEFs) were prepared from E14.5 embryos obtained from Vasa::RFP female transgenic mice^45^ mated with Oct4ΔPE-GFP male transgenic mice^46^. Those mice were maintained in C57Bl/6 background. The mice were kept and bred in an environmentally controlled and specific pathogen-free facility, the Animal Unit of the Institute of Development, Aging and Cancer (Tohoku University), in accordance with the approved guidelines for experimental animals defined by the facility. Animal protocols were reviewed and approved by the Tohoku University Animal Studies Committee. MEFs were cultured in high glucose Dulbecco’s Modified Eagle’s Medium (Gibco) supplemented with 10% fetal bovine serum FBS (Gibco) or Stem Pro 34 SFM (Gibco) supplemented with Stem Pro Nutrient supplement (Gibco), 10% knockout serum replacement (Gibco), 1% non-essential amino acid (Gibco), 1% Sodium pyruvated (Gibco), 4 mM L-glutamine (Gibco), 100 μM β-mercaptoethanol (SIGMA), 1000 Units/ml Recombinant Human Leukemia Inhibitory Factor (Millipore), 10 ng/ml basic Fibroblast Growth Factor (SIGMA), 10 ng/ml Recombinant Rat Glial Cell Line-derived Neurotrophic Factor (Gibco), 100 Units/ml Penicillin (SIGMA), and 100 μg/ml Streptomycin (SIGMA). MEFs were maintained at 37 °C in 5% CO_2_. The MEF isolation was carried out in accordance with the approved guidelines. VR15 ESCs were cultured as described previously^40^.

### PGCLC culture condition

2000 cells of the treated MEFs were cultured in a Lipidure-Coat 96-well plate (Thermo Fishers) in Glasgow’s Modified Eagle’s Medium (Gibco) supplemented with15% knockout serum replacement (Gibco), 1% non-essential amino acid (Gibco), 1% Sodium pyruvated (Gibco), 4 mM L-glutamine (Gibco), 180 μM β-mercaptoethanol (SIGMA), 500 ng/ml BMP4 (R&D), 500 ng/ml BMP8a (R&D), 100 ng/ml SCF (R&D), 1000 U/ml LIF (Millipore) and 50 ng/ml EGF (R&D) as previously described^44^.

### Plasmids

The Yamanaka factor (Oct4, c-Myc, Klf4, Sox2: OCKS) expressing tandem vector (pPB-CAG-OCKS) were provided from PiggyBac Transposes Resources of Wellcome trust Sanger Institute via Dr. T. Noce. The Oct4 and Sox2 expressing vectors (PB-hCMV*1-Oct3/4-pA and PB-hCMV*1-Sox2-pA) were constructed by transferring these ORFs from pPyCAG-Oct3/4-IP and pPyCAG-Sox2^47^ to pPB-hCMV*1-pA vector^48^.

### Transfection

Plasmids and siRNAs were transfected into cells using Lipofectamine 2000 (Invitrogen) via the reverse method described in the manufacturer’s instructions. Transfection in the 24-well plate format was carried out as follows. Lipofectamine 2000 (2 μl), plasmids (0.4 μg) and siRNAs (20 pmol) were diluted with 100 μl Opti-MEM and incubated for 20 min at room temperature. Then, 50000 cells suspended in 500 μl of growth medium were added to each Lipofectamine/Plasmids/siRNA mixture and were mixed and plated into each well of a 24-well plate. The cells were incubated for 24 hr and were fed with growth medium with/without small-molecule compounds. All siRNAs were designed by QIAGEN. IDs of the siRNAs used in this study are Mm_Max_5, Mm_LOC100044129_1, Mm_Ezh2_6, Mm_Atf7ip_3 and Mm_Dnmt1_2. AllStars Negative control siRNA (QIAGEN) was used as the non-silencing control siRNA. Details of the small-molecule compounds are shown in Supplementary Table S1.

### RNA preparation and real-time PCR

Total RNA samples were purified using the RNeasy Plus mini kit (QIAGEN) according to the manufacturer’s instructions. RNAs were reverse-transcribed using SuperScript III (Invitrogen) and random primers (Promega). Expression levels of germ cell-specific genes or somatic genes were quantified using the SYBR Green Master Mix (Applied Biosystems) with the primers shown in Supplementary Table S2 or via TaqMan Gene expression assays (Applied Biosystems). PCR signals were detected using CFX Connect (Bio-Rad). *Ppia*, *Arbp*, or *Gapdh* was used as an internal control. The TaqMan probes used in this study were *Dppa3* Mm00836373_g1, *Sycp1* Mm01298009_m1, and Gapdh 4352932E.

### Microarray analysis

Total RNA (100 ng) from each sample was analyzed. Samples were prepared using the Agilent Low Input Quick Amp Labeling Kit; probes were hybridized onto the Agilent Whole Mouse Genome Oligo DNA Microarray kit Ver 2.0 (Agilent) according to the manufacturer’s instructions. The microarrays were scanned using the Agilent DNA microarray scanner (Agilent). Each cell-type was analyzed in three biological replicate. Multiple testing corrections were performed with the Benjamini-Hochberg false-discovery rate correction.

### Immunofluorescence

For immuno-staining of Dazl protein, cells were fixed with 4% paraformaldehyde in PBS for 1 hr at room temperature, permeabilized with 0.3% Triton-X in PBS for 15 min. For immuno-staining of Vasa protein, cells were fixed with 4% paraformaldehyde in PBS for 1 hr at room temperature, incubated with 0.2 M Glycine for 10 min at room temperature. Then the cells were re-fixed with 100% methanols for 10 min at −20 °C, and were incubated with blocking solution (10% FBS, 1% BSA, 0.1% Triton-X in PBS) for 1 hr at 4 °C. The cells were then incubated with primary antibody (rabbit anti-Dazl, Abcam; rabbit anti-Vasa^49^) in the blocking solution overnight at 4 °C. The cells were then washed and incubated with secondary antibody (Alexa Fluor dye-conjugated secondary antibodies, Invitrogen) and 3 μg/ml^−1^ 4′,6-diamidino-2-phenylindole (DAPI) in blocking solution for 1 hr at room temperature. The cells were washed again and mounted with VECTASHIELD (Vectro Laboratories). Stained cells were observed under a Leica AF6000 fluorescence microscope, and were then analyzed with image J. The immune-staining of Sycp3 protein were performed as described previously using anti-Sycp3 (Abcam)^40^.

### Bisulphite sequencing

Genomic DNA was extracted using QIAGEN DNeasy Blood & Tissue kits or QIAGEN All prep DNA/RNA Micro kits and converted with sodium bisulphite using the EZ DNA Methylation-Direct Kit (Zymo Research) according to manufacturer’s instructions. The targeted regions of *Dazl*, *Tex19.1* and *Sycp1* were amplified from bisulphite-converted DNAs using BIOTAQ HS DNA Polymerase (BIOLINE). The sequences of the PCR primers used for this assay are listed in Supplementary Table S2. The PCR products were cloned into respective pGEM-T easy vectors (Promega) and were sequenced using the BigDye Terminator v1.1 Cycle Sequencing Kit (Applied Biosystems).

### Chromatin immunoprecipitation (ChIP)

ChIP experiments were carried out as described previously^50^ with some modifications. In brief, 5 μg of antibodies were bound to Dynabeads Protein G (Invitrogen) for normal IgG (CST), anti-H3K4me3 (Abcam), and anti-H3K27me3 (Millipore) overnight at 4 °C. Each cell suspension was then fixed with 1% formaldehyde in DPBS (Invitrogen) for 10 min. After adding 1.25M glycine, the cross-linked cells were washed, collected in pellets by centrifugation, and flash frozen with liquid nitrogen. The cells were lysed in SDS lysis buffer, and genomic DNA was sheared by sonication (Branson sonifier 250). After centrifugation, each cleared lysate was incubated with antibody-bound Dynabeads overnight at 4 °C. The beads were washed, and the chromatin was eluted and subjected to reverse cross-linking. DNA was purified using the QIAGEN PCR purification kit, and analyzed via real-time PCR using Power SYBR Green PCR master mix (Applied Biosystems) and primers that spanned the transcriptional start sites of the genes of interest. The primer sequences used for these assays are listed in Supplementary Table S2.

### Alkaline phosphatase staining

For alkaline phosphatase (ALP) staining, cells were fixed with 4% paraformaldehyde in PBS for 15 min at room temperature, and then incubated with staining solution (25 mM Tris-Malate pH9.0 (Wako), 0.4 mg/ml Sodium α naphthyl phosphate (SIGMA), 1 mg/ml Fast Red TR solt (SIGMA) in water) for 30 min at room temperature. The cells were washed by PBS, then were observed under a Nikon TE300 microscope.

## Additional Information

**How to cite this article**: Sekinaka, T. *et al*. Selective de-repression of germ cell-specific genes in mouse embryonic fibroblasts in a permissive epigenetic environment. *Sci. Rep.* **6**, 32932; doi: 10.1038/srep32932 (2016).

## Supplementary Material

Supplementary Information
