# Supplementary material for: Selective de-repression of germ cell-specific genes in mouse embryonic fibroblasts in a permissive epigenetic environment
Source: Sci Rep. 2016 Sep 9;6:32932. doi: 10.1038/srep32932 (PMC5016969; doi:10.1038/srep32932)
Supplement: Supplementary Information [file srep32932-s1.pdf]

Selective de-repression of germ cell-specific genes in mouse embryonic fibroblasts in a permissive epigenetic environment

Tamotsu Sekinaka, Youhei Hayashi, Toshiaki Noce, Hitoshi Niwa, Yasuhisa Matsui

Supplementary Figure S1 A flow chart showing our strategy to explore conditions for inducing germ cell-specific genes

Supplementary Figure S2 Knockdown efficiency of Max, Ezh1, Ezh2 and Dnmt1 by RNAi

The expression of germ cell-specific genes was quantified by real-time PCR in MEFs after 2 days in culture under OCKS+Ctrl siRNA, OCKS+*Max*-KD, OCKS+Ezh1 KD, OCKS+Ezh2 KD or OCKS+Dnmt1 KD conditions. The expression in MEFs with OCKS+Ctrl siRNA was set as 1.0. The data were obtained by a single experiment.

Supplementary Figure S3 Quantification of germ cell-specific genes expression in OCKS+*Max*-KD with or without VAP and ALK5i conditions

The expression of germ cell-specific genes was quantified by real-time PCR in MEFs after 2 days in culture under OCKS+Ctrl siRNA or OCKS+*Max*-KD conditions with or without VPA and ALK5i (VA5). The expression in MEFs with OCKS+*Max*-KD was set as 1.0. Error bars, S.E. of three biological replicates, *p<0.05, **p<0.01, ***p<0.001 (Student's t-test).

Supplementary Figure S4 The expression of Vasa in MEFs following inhibition of epigenetic modifications by RNAi or by small-molecule compounds

(a) The expression of *Vasa* in MEFs after 2 days in culture in OCKS+Ctrl siRNA, OCKS+*Ezh1*- and *Ezh2*-KD, OCKS+*Max*-, *Ezh1*- and *Ezh2*-KD or OCKS+*Max*- and *Atf7ip*-KD conditions was quantified by real-time PCR. The expression in MEFs with OCKS+Ctrl siRNA was set as 1.0.

(b) The expression of *Vasa* in MEFs after 2 days in culture in OCKS+Ctrl siRNA, OCKS+*Max*- and *Atf7ip*-KD, or OCKS+*Max*- and *Atf7ip*-KD with VPA and ALK5i (VA5) conditions. The expression in MEFs with OCKS+Ctrl siRNA was set as 1.0.

(c) The expression of *Vasa* in MEFs after 2 days in culture in OCKS with or without chem+VA5. DMSO was added to control cells. The expression in MEFs in OCKS+DMSO condition was set as 1.0. The data were obtained by a single experiment.

Supplementary Figure S5 The germ cell-specific genes were significantly upregulated in OS+chem+VA5+*Dnmt1*-KD condition

(a) The expression of germ cell-specific genes in MEFs after 2 days in culture in OCKS+Ctrl siRNA, OCKS+*Max*- and *Atf7ip*-KD+VA5, or OCKS+chem+VA5, with inhibition of DNA methylation by *Dnmt1*-KD or by 5-Aza-cytidine (Aza) conditions. The expression in MEFs with OCKS+chem+VA5+*Dnmt1*-KD quantified by real-time PCR was set as 1.0.

(b) The expression of somatic genes (*Hoxa1* and *Hoxb1*) in MEFs 2 days in culture in OCKS+Ctrl siRNA or OCKS+chem+VA5+*Dnmt1*-KD conditions. The expression in MEFs with OCKS+chem+VA5+*Dnmt1*-KD was set as 1.0.

Error bars: S.E. of three biological replicates, *$p<0.05$, **$p<0.01$, ***$p<0.001$ (Student's t-test).

Supplementary Figure S6 Change of Vasa expression in MEFs in OCKS+chem+VA5+Dnmt1 KD condition after prolonged culture

The expression of *Vasa* was quantified by real-time PCR in MEFs after transfection of the expression vector encoding the Yamanaka factors (*Oct4, c-Myc, Klf4, Sox2*) with or without tranylcipromine, BIX-01294, DZNep (chem), VPA, ALK5i (VA5), and Dnmt1 Knocked-Down (KD) (OCKS+chem+VA5+Dnmt1 KD, or OCKS+Ctrl siRNA) after 2, 4, 6 days in culture. The expression level of Vasa in MEFs with OCKS+chem+VA5+Dnmt1 KD was set as 1.0. The data were obtained by a single experiment.

Supplementary Figure S7 The expression of Dazl protein was significantly induced in MEFs in the OCKS+chem+VA5+*Dnmt1*-KD condition

(a) Immuno-fluorescence staining of MEFs after 4 days in culture in OCKS+chem+VA5+*Dnmt1*-KD or OCKS+Ctrl siRNA conditions using anti-Dazl antibody. The shown data are representative of data from three independent experiments. Red: anti-Dazl, Blue: DAPI, Scale bar: 200 μm.

(b) The ratios of number of Dazl-positive cells in DAPI-positive cells. Dazl- and DAPI-positive cells were estimated by image J. Error bars: S. E. of three biological

replicates, *p<0.05.

Supplementary Figure S8 Transcriptome analysis of MEFs cultured in OCKS+chem+VA5+*Dnmt1*-KD conditions

(a) The ratios of number of up- and downregulation genes in MEFs after 4 days in OCKS+chem+VA5+*Dnmt1*-KD condition compared with in control MEFs. Genes with at least two-fold changes in expression (p<0.001) were analyzed.

(b) The ratios of upregulated genes annotated with particular GO terms.

(c) The ratios of downregulated genes annotated with particular GO terms.

(d) Relationship of upregulated genes in E13.5 male PGCs and in the treated MEFs compared with in control MEFs.

(e) GO analysis in commonly upregulated genes in E13.5 male PGCs and in the treated MEFs compared with in control MEFs. The GO terms with corrected p-value <0.05 are shown.

The array data were obtained from three biological replicates.

Supplementary Figure S9 The expression of Dazl, Tex19.1 and Sycp1 in the OS+chem+VA5+*Dnmt1*-KD condition

Scatter plot of meiosis-related genes at least two-fold upregulated in MEFs in OS+chem+V.A+*Dnmt1*-KD condition compared with in control MEFs.

Supplementary Figure S10 The expression of germ cell-specific genes in E13.5 male PGCs

(a) The expression of Vasa in E13.5 male (♂) PGC and in MEFs after 2 days in culture with OCKS+*Max*- and *Atf7ip*-KD+VA5 or OCKS+chem+VA5 treatment. The expression in MEFs in OCKS+*Max*- and *Atf7ip*-KD+VA5 or OCKS+chem+VA5 condition was set as 1.0. The data were obtained by a single experiment.

(b) The expression of Dazl, Tex19.1 and Sycp1 in E13.5 ♂ PGC and in MEFs after 4 days in culture with OS+chem+VA5+Dnmt1 KD treatment. The expression in MEFs in OS+chem+VA5+Dnmt1 KD condition was set as 1.0. Error bars: S. E. of three biological replicates, ***p<0.001 (Student's t-test).

The expression of germ cell-specific genes was quantified by real-time PCR.

Supplementary Figure S11 The effect of OS for the expression of Dazl, Tex19.1 and Sycp1 in the ALK5i, DZNep, Dnmt1 KD condition

The expression of Dazl, Tex19.1 and Sycp1 in MEFs after 4 days in culture under ALK5i+DZNep+*Dnmt1*-KD or OS+ALK5i+DZNep+*Dnmt1*-KD conditions. The expression in MEFs in the OS+ALK5i+DZNep+*Dnmt1*-KD condition was set as 1.0. The expression of Dazl, Tex19.1 and Sycp1 was quantified by real-time PCR. The data were obtained by a single experiment.

Supplementary Figure S12 Changes of histone modification status in the OS+chem+VA5+*Dnmt1*-KD condition

ChIP analysis of the promoter regions of *Dazl, Tex19.1*, and *Sycp1* for H3K4me3 and H3K27me3 in MEFs 4 days after the OS+chem+VA5+*Dnmt1*-KD treatment or in control MEFs. ChIP using normal IgG was used as a negative control. Levels of H3K4me3 or H3K27me3 were determined by real-time PCR, and the percentages of values relative to those for input chromatin are shown. Other data from two independent experiments are also shown in Fig. 6b.

Supplementary Figure S13 Immuno-staining of Vasa protein in MEFs cultured with OS+chem+VA5+Dnmt1 KD

Immuno-fluorescence staining of MEFs after 4 days in culture in OCKS+chem+VA5+*Dnmt1*-KD or OCKS+Ctrl siRNA conditions using anti-Vasa antibody. Red: anti-Vasa, Blue: DAPI, Scale bar: 100 μm. VR15 ES cells after *Max* knock-down (*Max*-KD VR15 ESC) were used for positive control of the immuno-staining.

Supplementary Figure S14 Immuno-staining of Sycp3 protein in OS+chem+VA5+Dnmt1 KD

Immuno-fluorescence staining of MEFs after 4 days in culture in OCKS+chem+VA5+*Dnmt1*-KD or OCKS+Ctrl siRNA conditions using anti-Sycp3 antibody. Red: anti-Sycp3, Blue: DAPI, Scale bar: 100 μm. VR15 ES cells after *Max* knock-down (*Max*-KD VR15 ESC) were used for positive control of the immuno-staining.

Supplementary Figure S15 The effect of OCKS+chem+VA5+*Dnmt1*-KD condition for reprogramming of MEFs to iPSCs.

(a) The expression of typical pluripotency markers, the Oct4ΔPE-GFP reporter and alkaline phosphatase (ALP) activity in MEFs cultured in the OCKS+Ctrl siRNA treatment for 15 days. The shown data are representative of three independent experiments. Scale bar: 200 μm.

(b) The number of Oct4ΔPE-GFP- or ALP-positive colonies in OCKS+Ctrl siRNA or OCKS+chem+VA5+Dnmt1 KD treatment for 15 days. Error bars: S. E. of three biological replicates, *$p<0.05$.

Supplementary Figure S16 The expression of *Blimp1* in OS+chem+VA5+Dnmt1 KD treated MEFs after cultured in the PGCLC culture condition

The MEFs cultured in OS+chem+VA5+Dnmt1 KD for 4 days were further cultured in the PGCLC culture condition for 6 days. The expression of *Blimp1* was quantified by real-time PCR. The expression in MEFs after 4 days in culture in OS+chem+VA5+Dnmt1 KD was set as 1.0. The data were obtained by a single experiment.

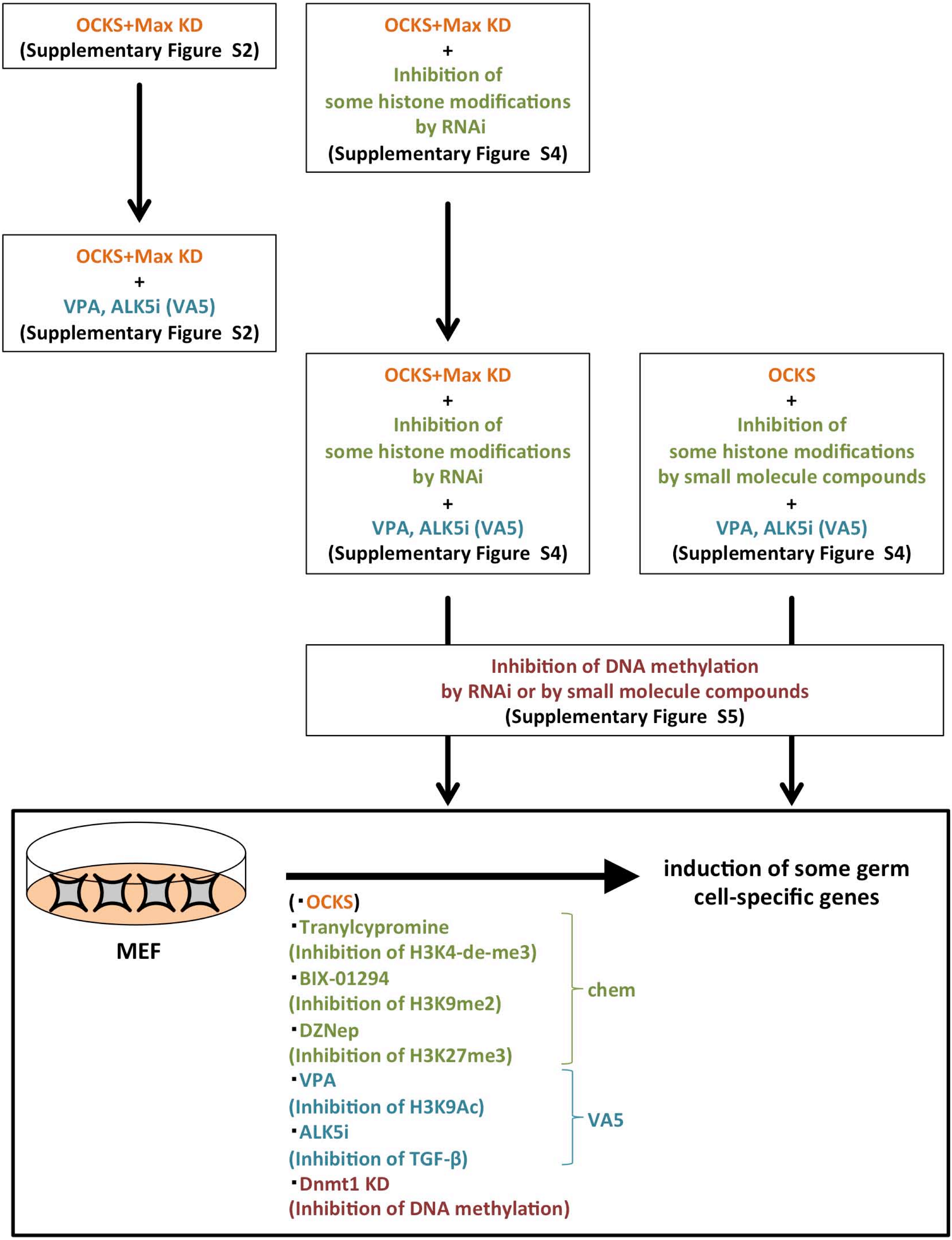

Supplementary Figure-S1 (Matsui)

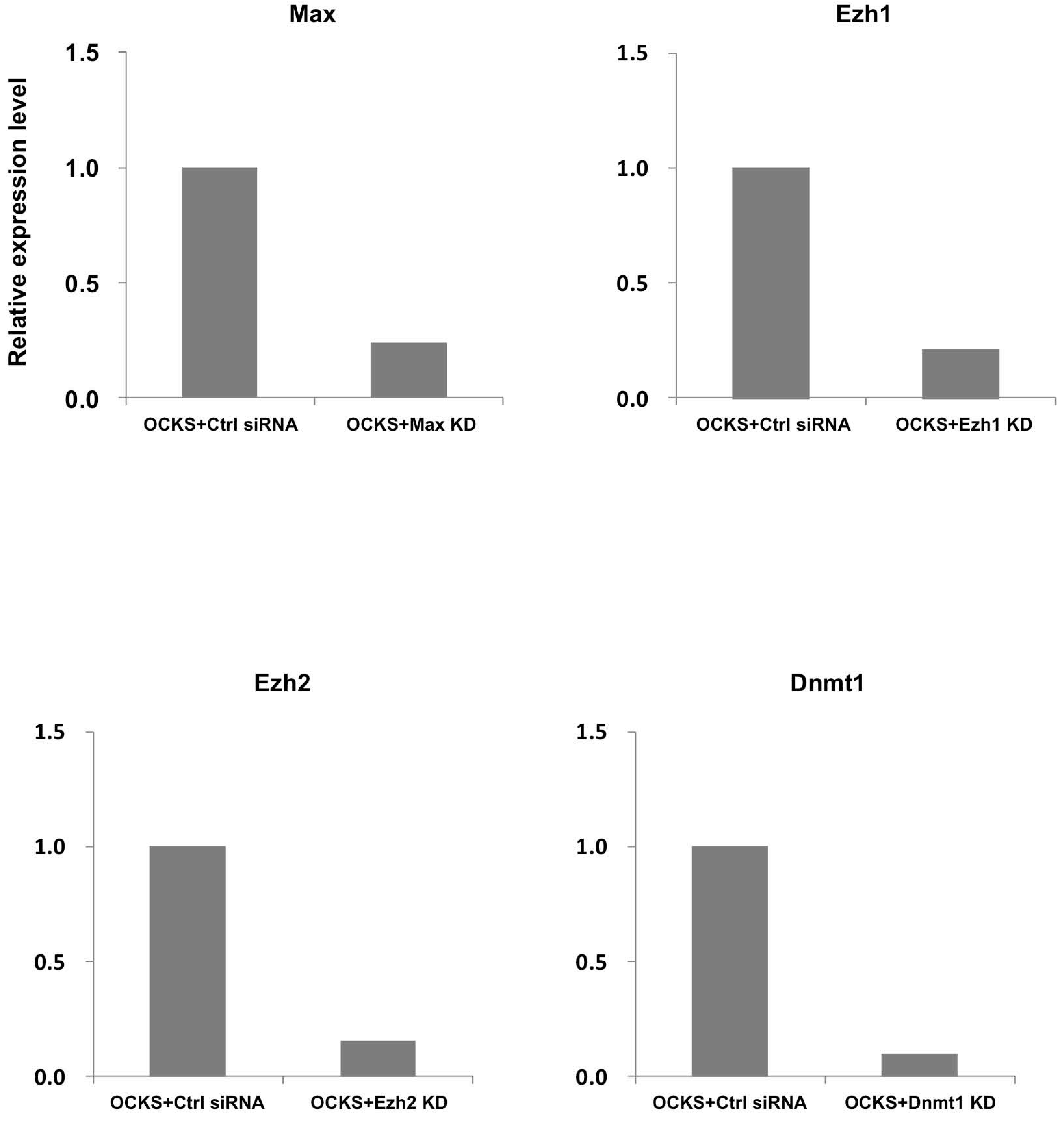

Supplementary Figure-S2 (Matsui)

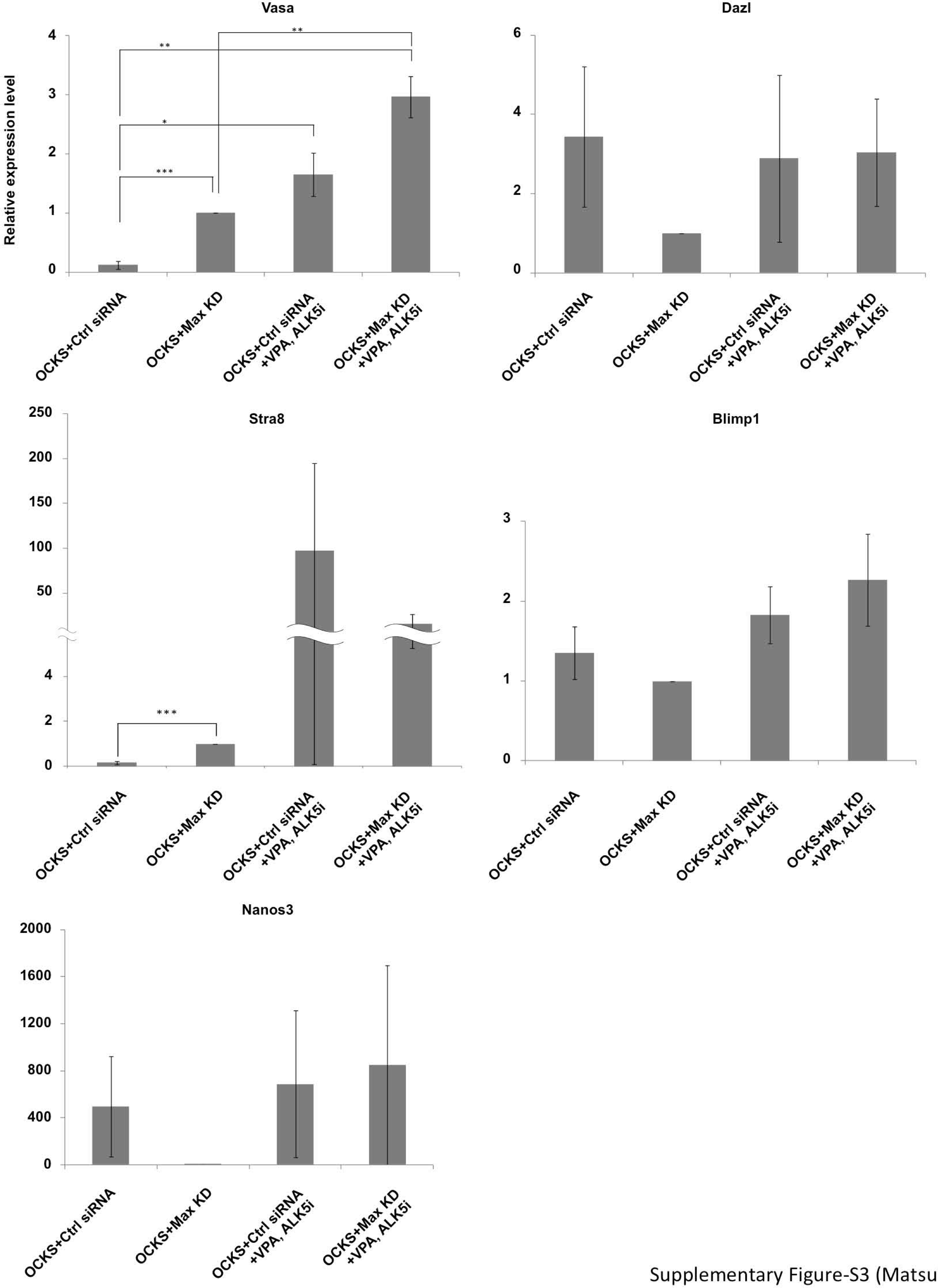

Supplementary Figure-S3 (Matsui)

a

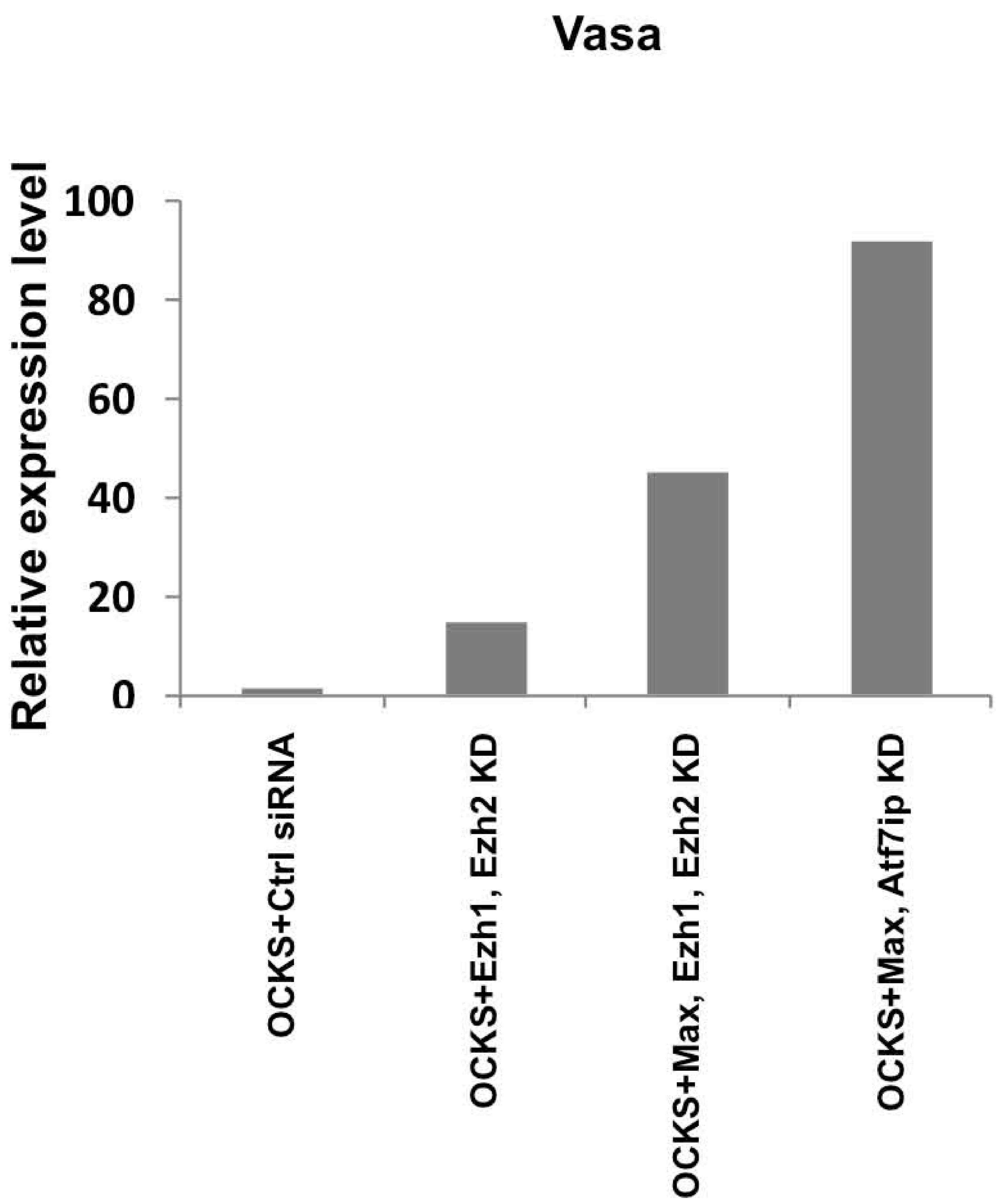

b

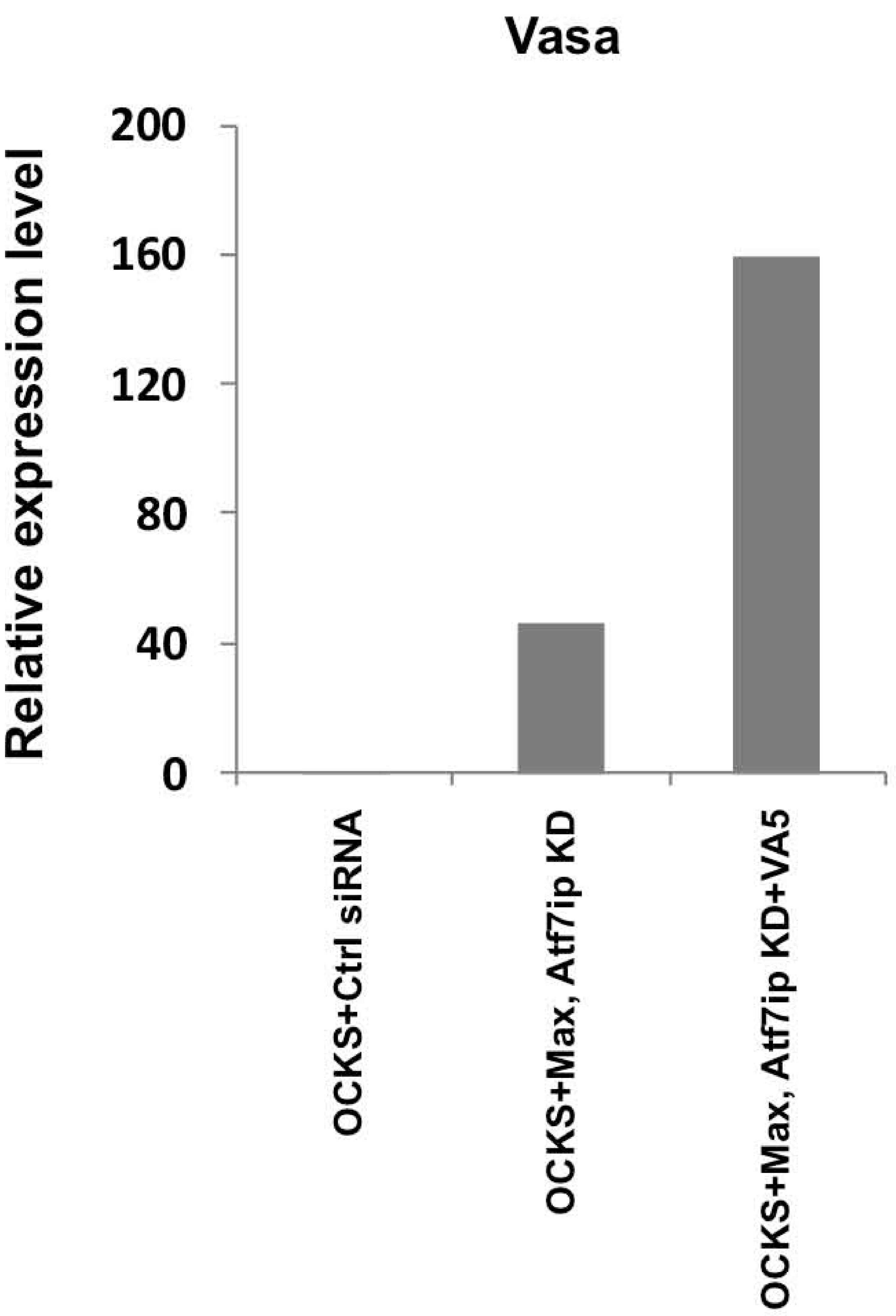

c

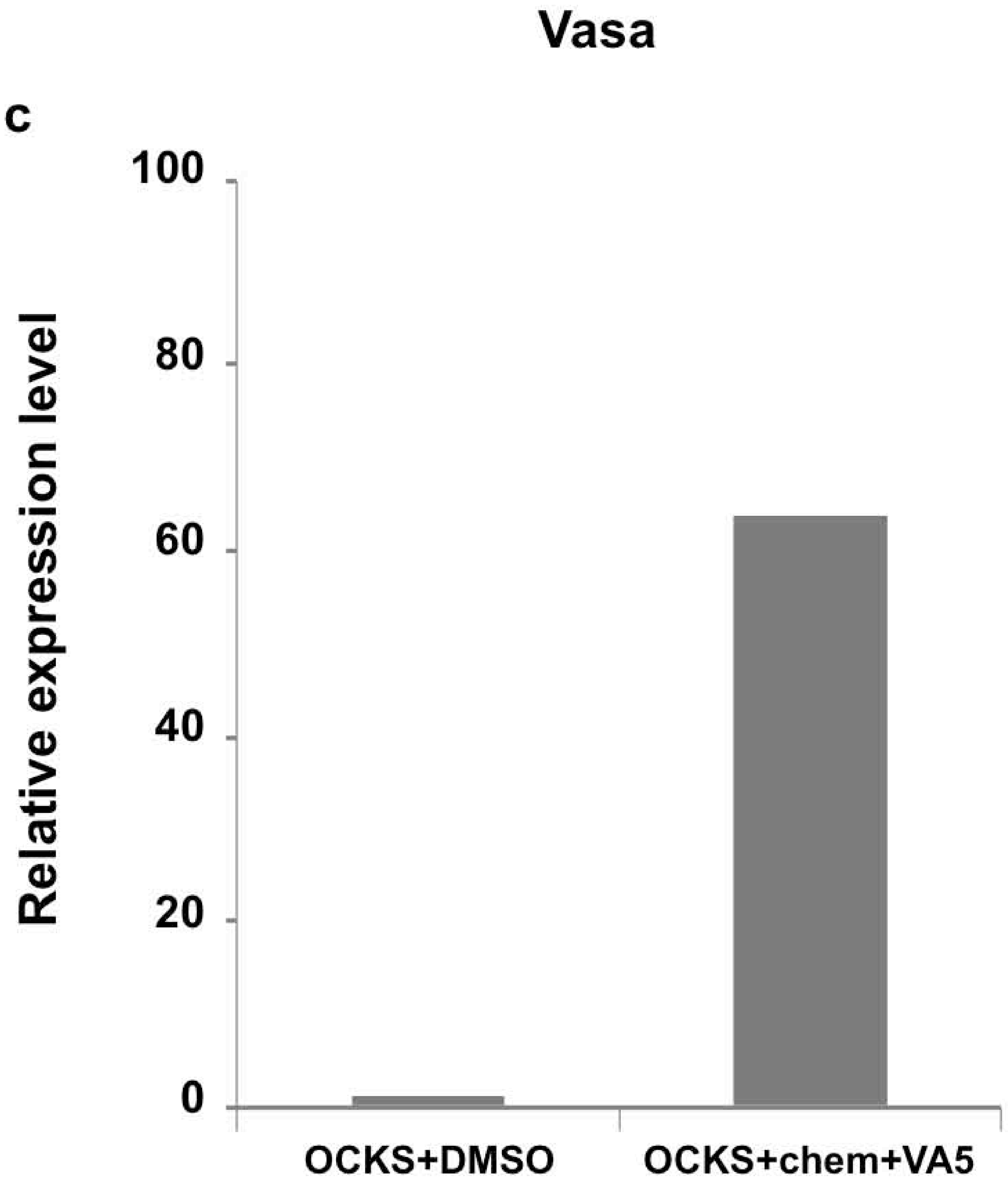

Supplementary Figure-S4 (Matsui)

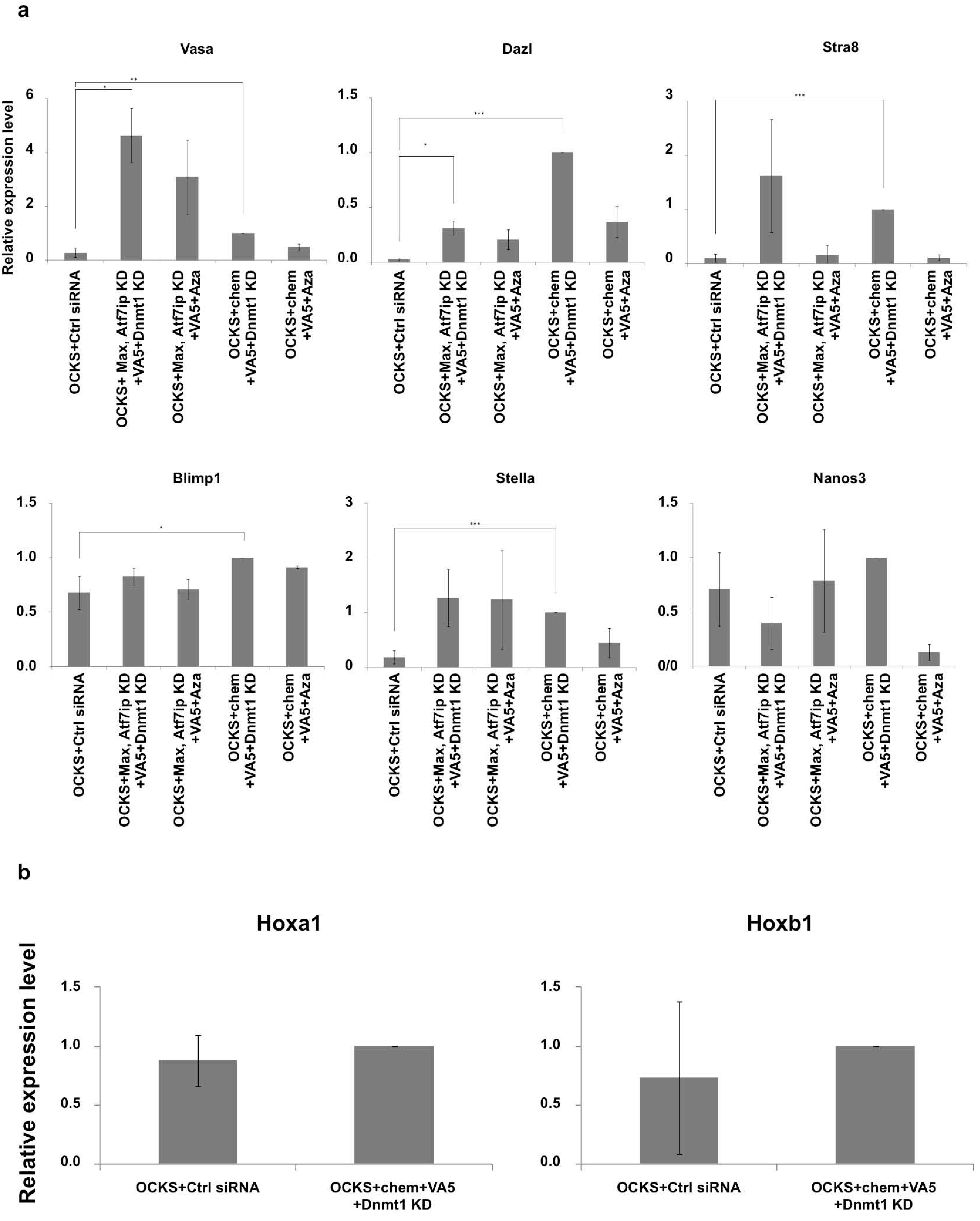

Supplementary Figure-S5 (Matsui)

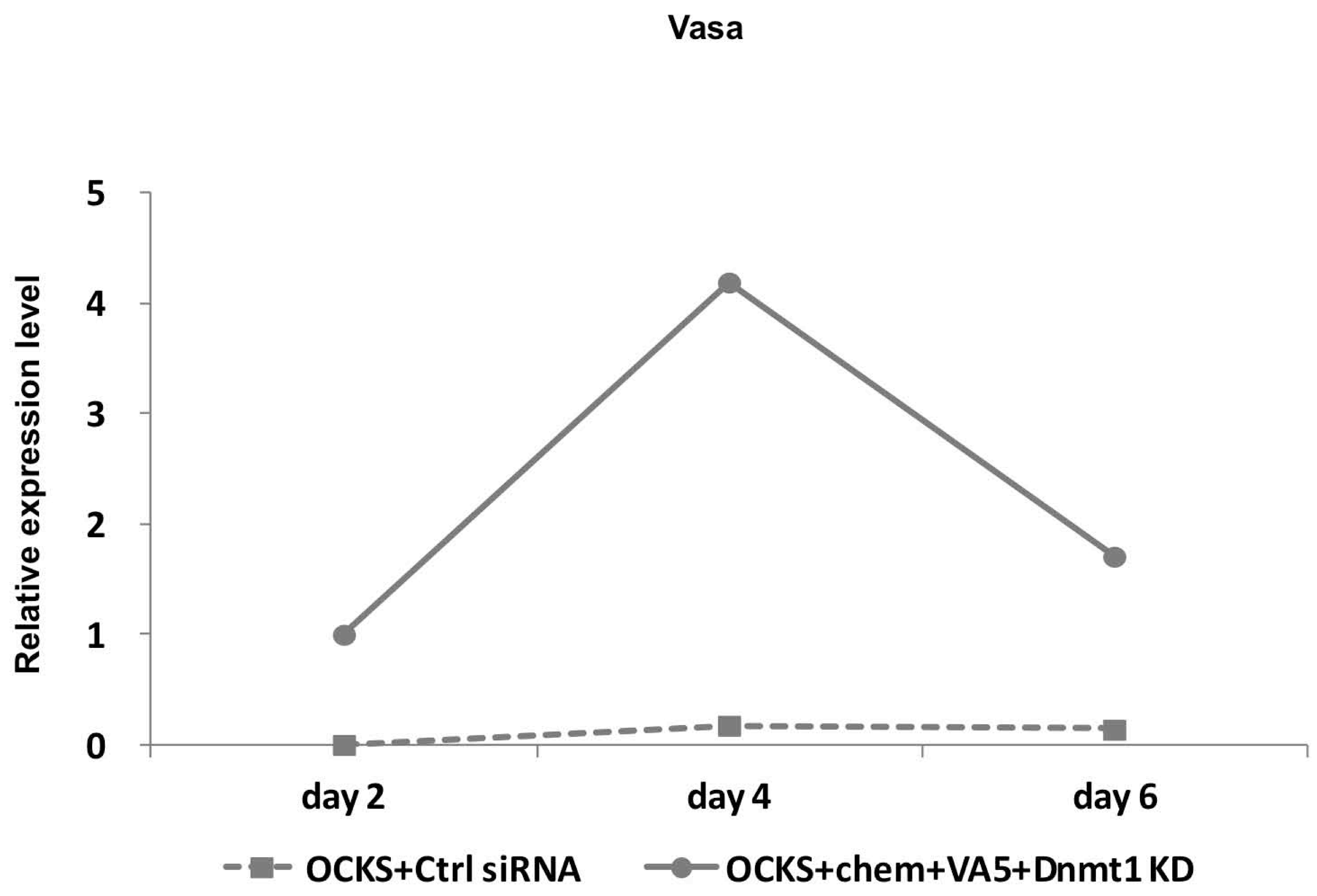

Supplementary Figure-S6 (Matsui)

a

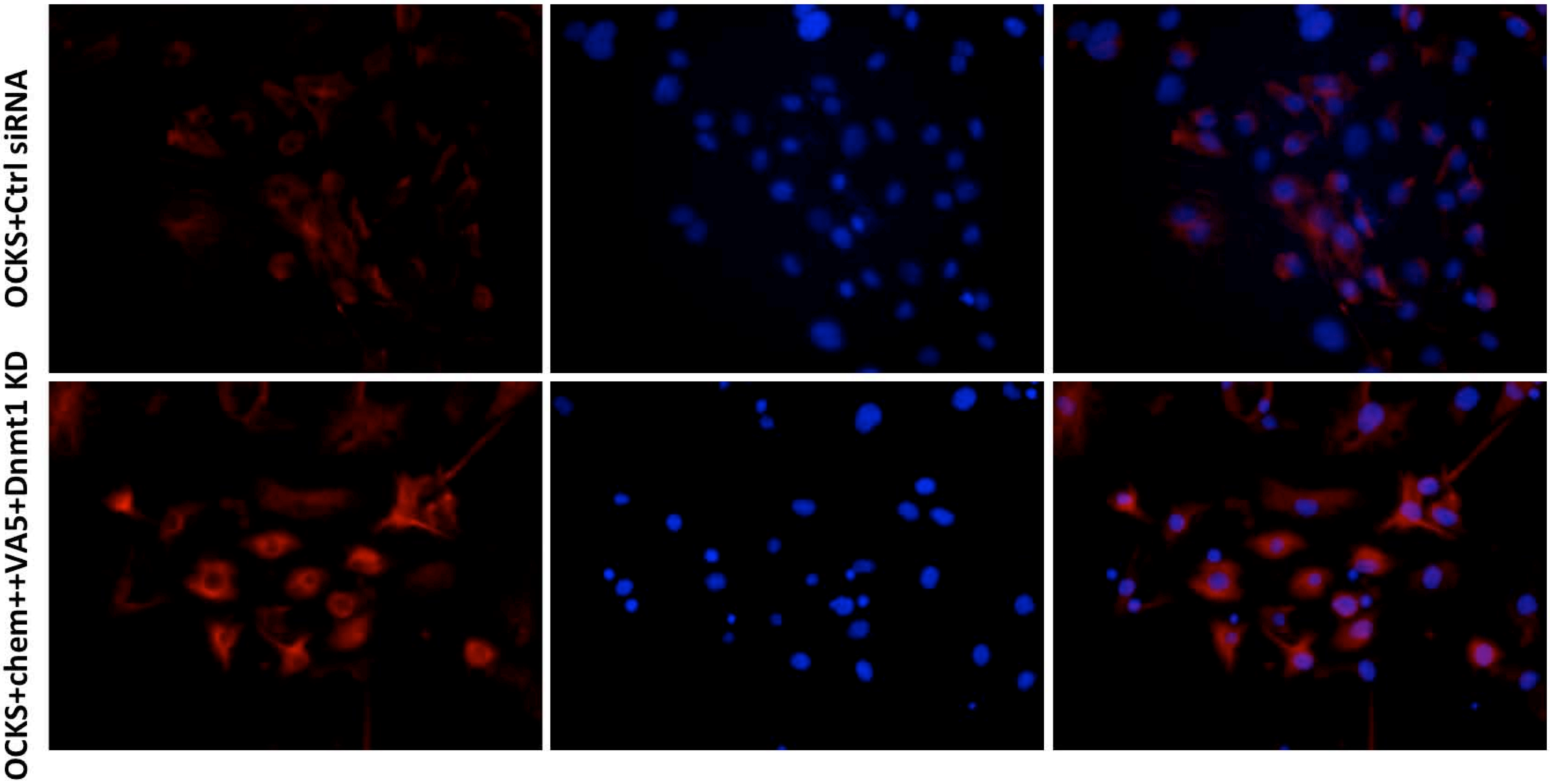

b

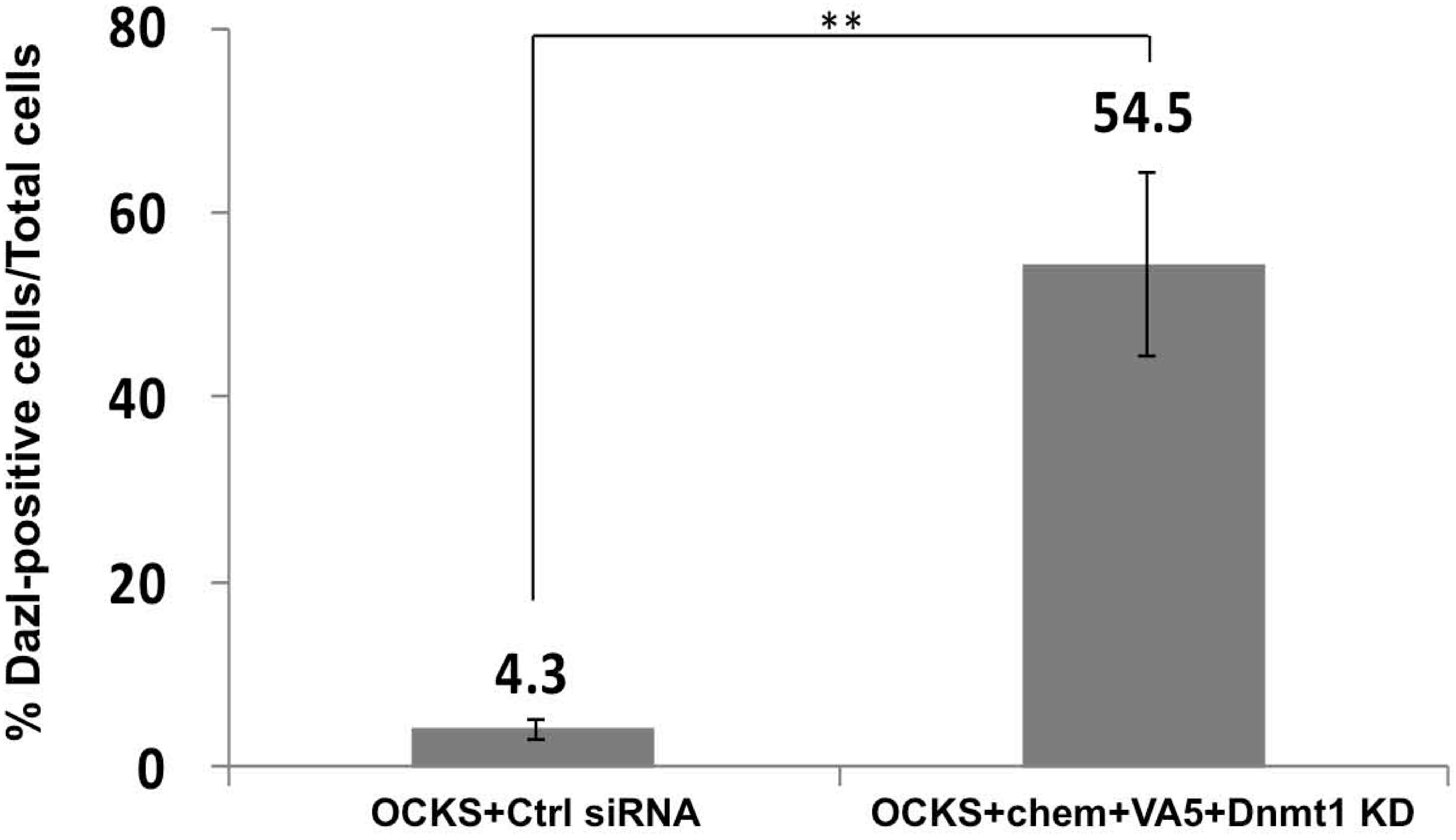

Supplementary Figure-S7 (Matsui)

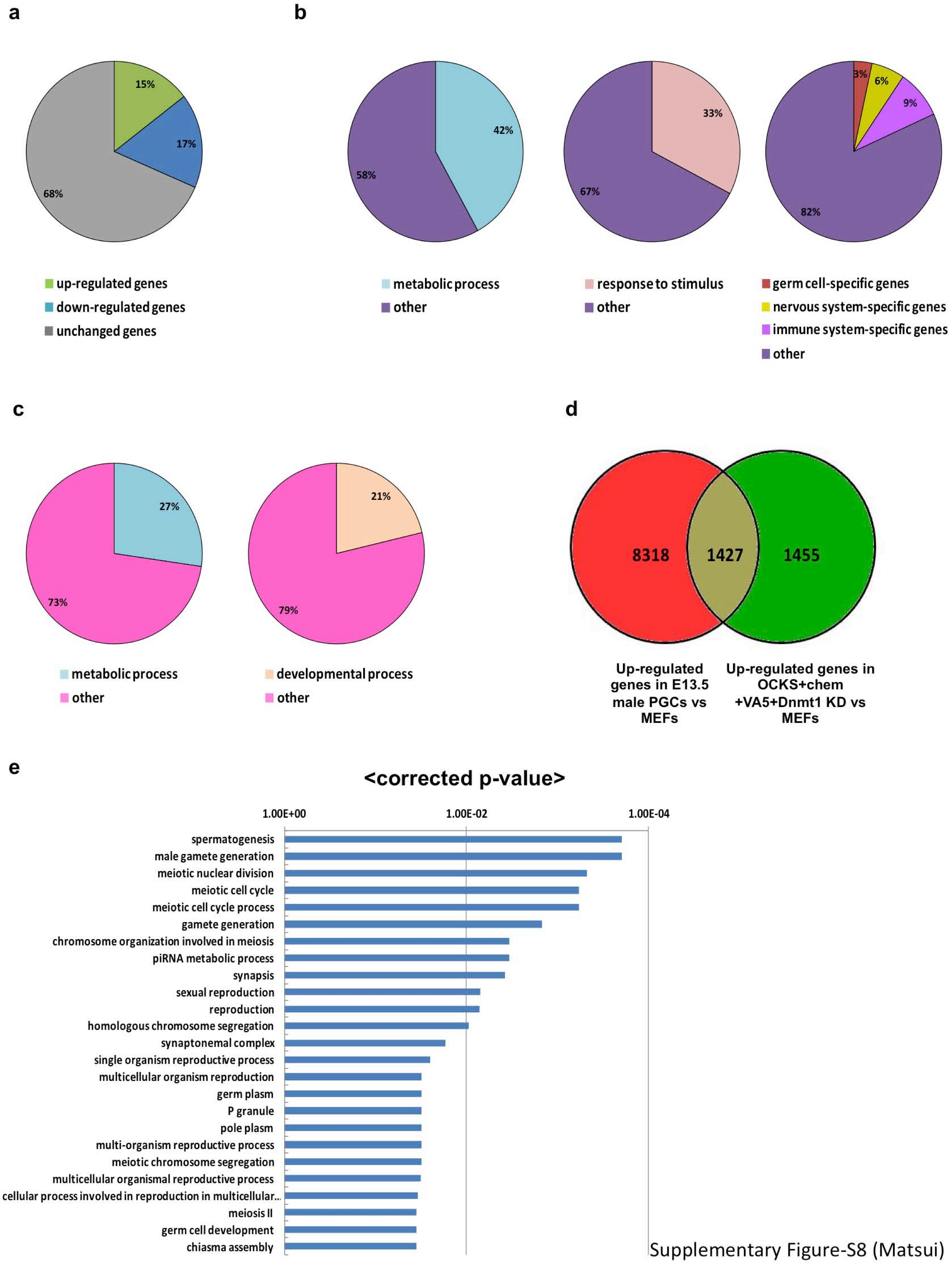

Supplementary Figure-S8 (Matsui)

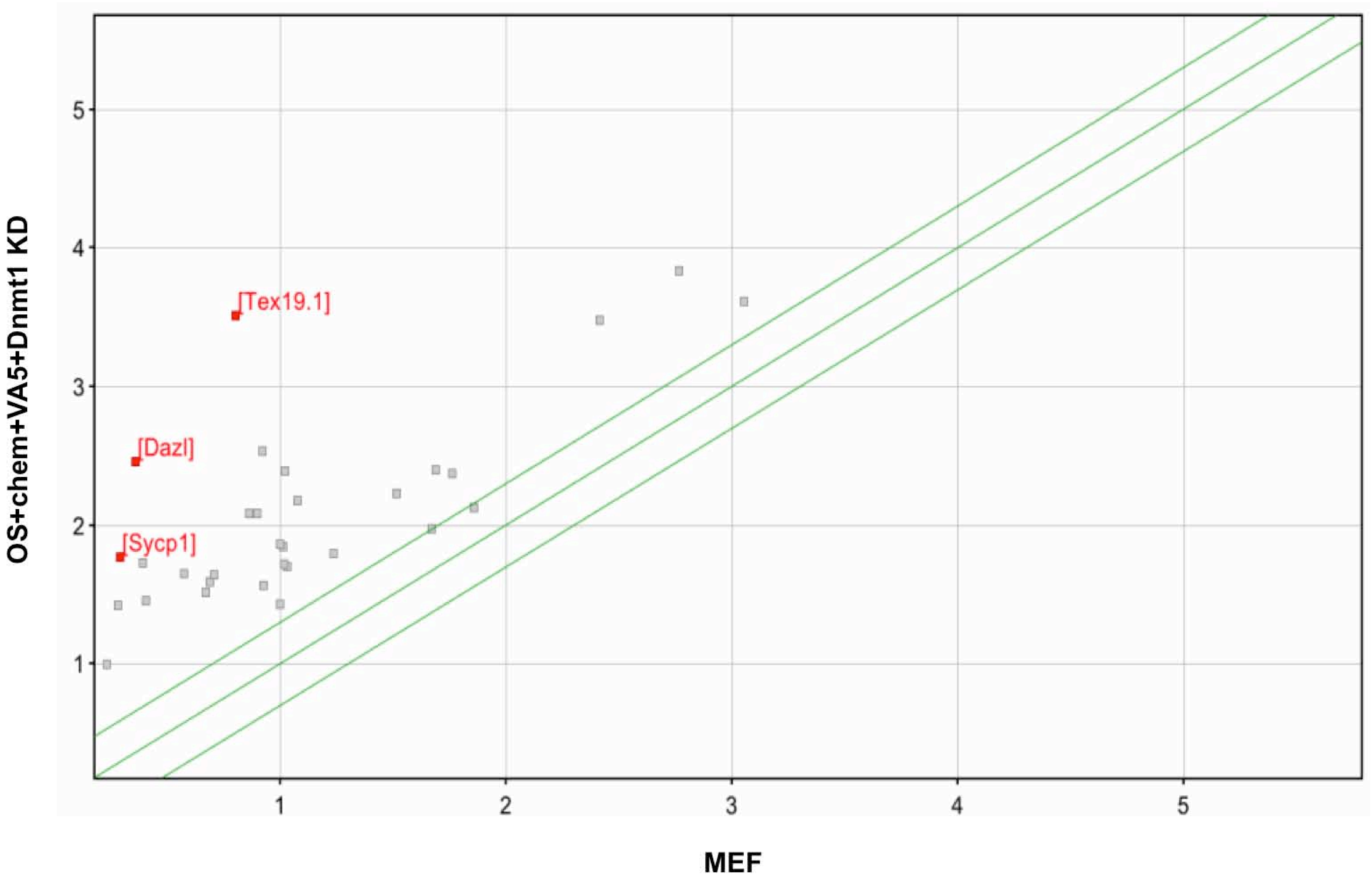

Supplementary Figure-S9 (Matsui)

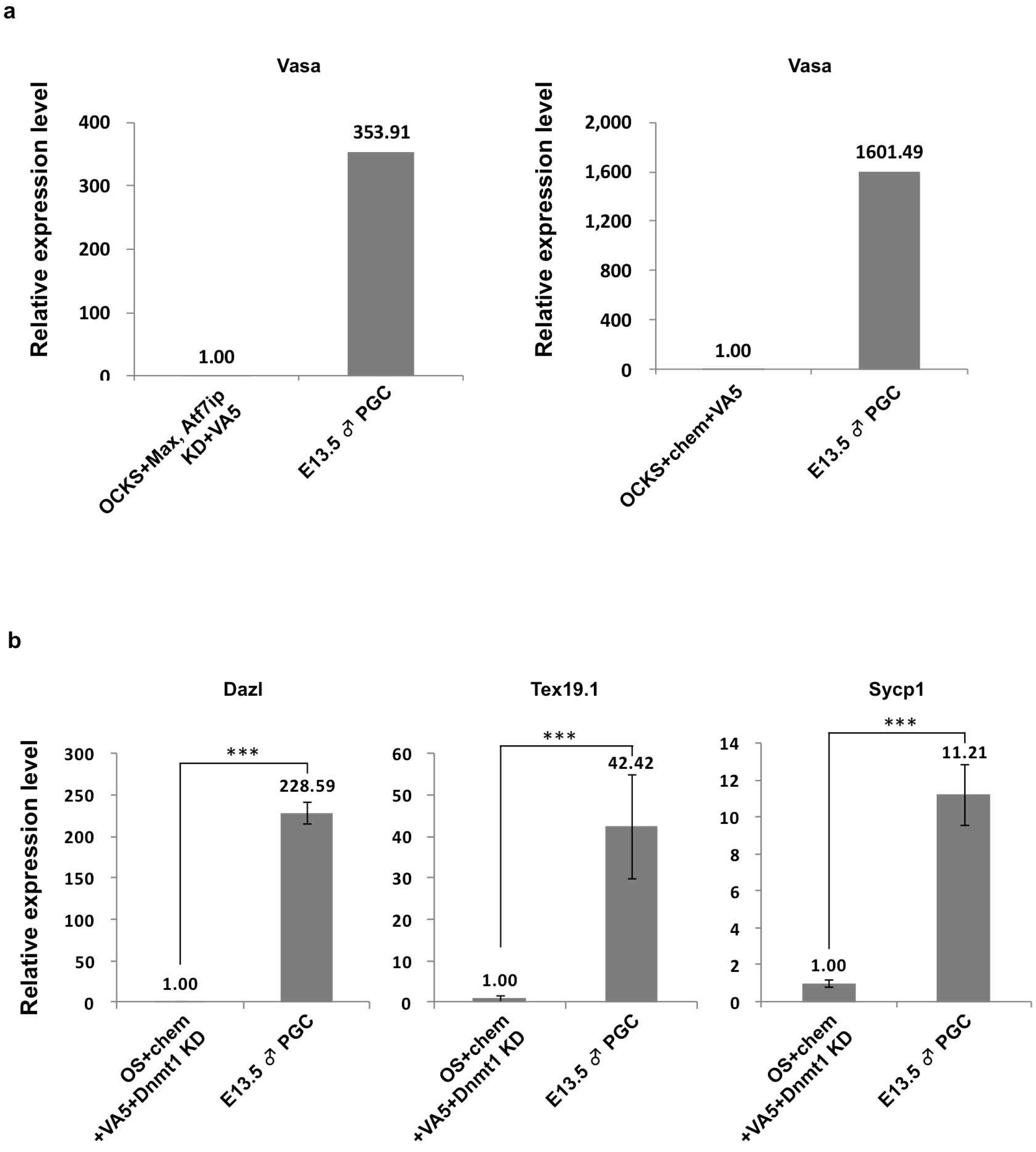

Supplementary Figure-S10 (Matsui)

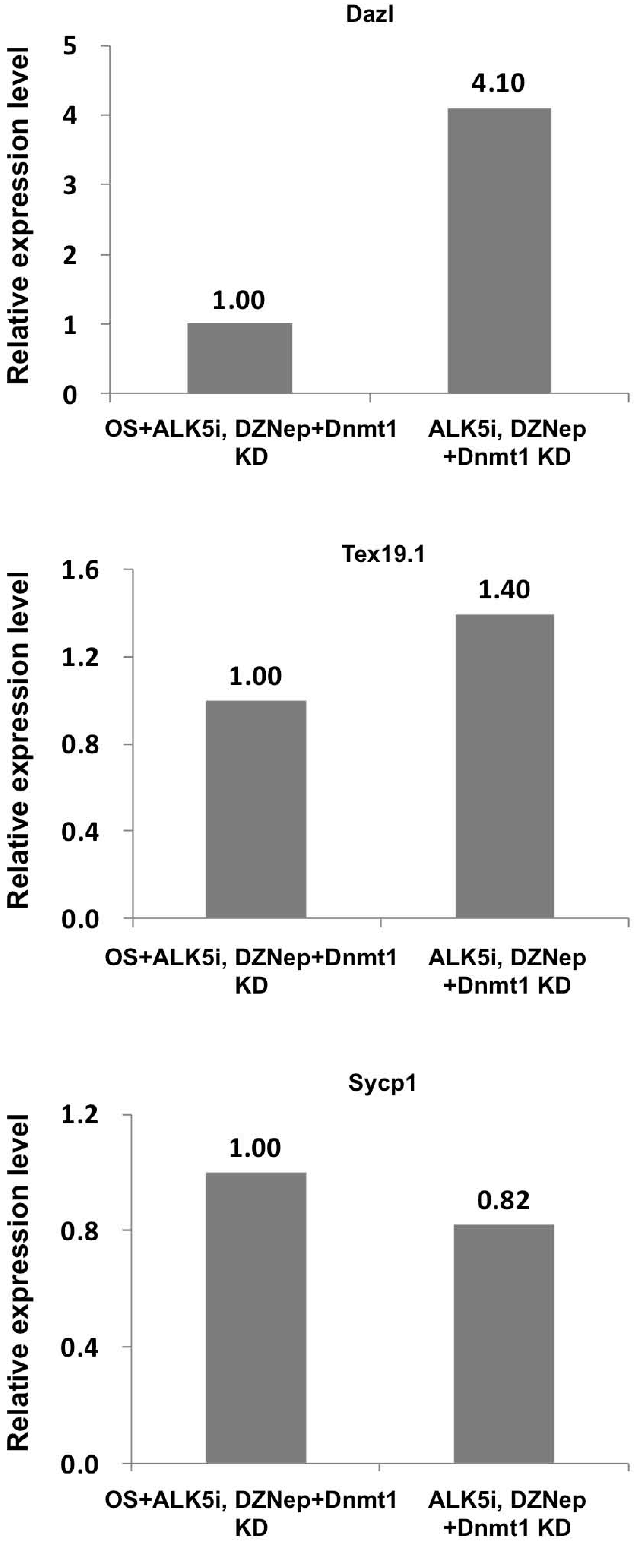

Supplementary Figure-S11 (Matsui)

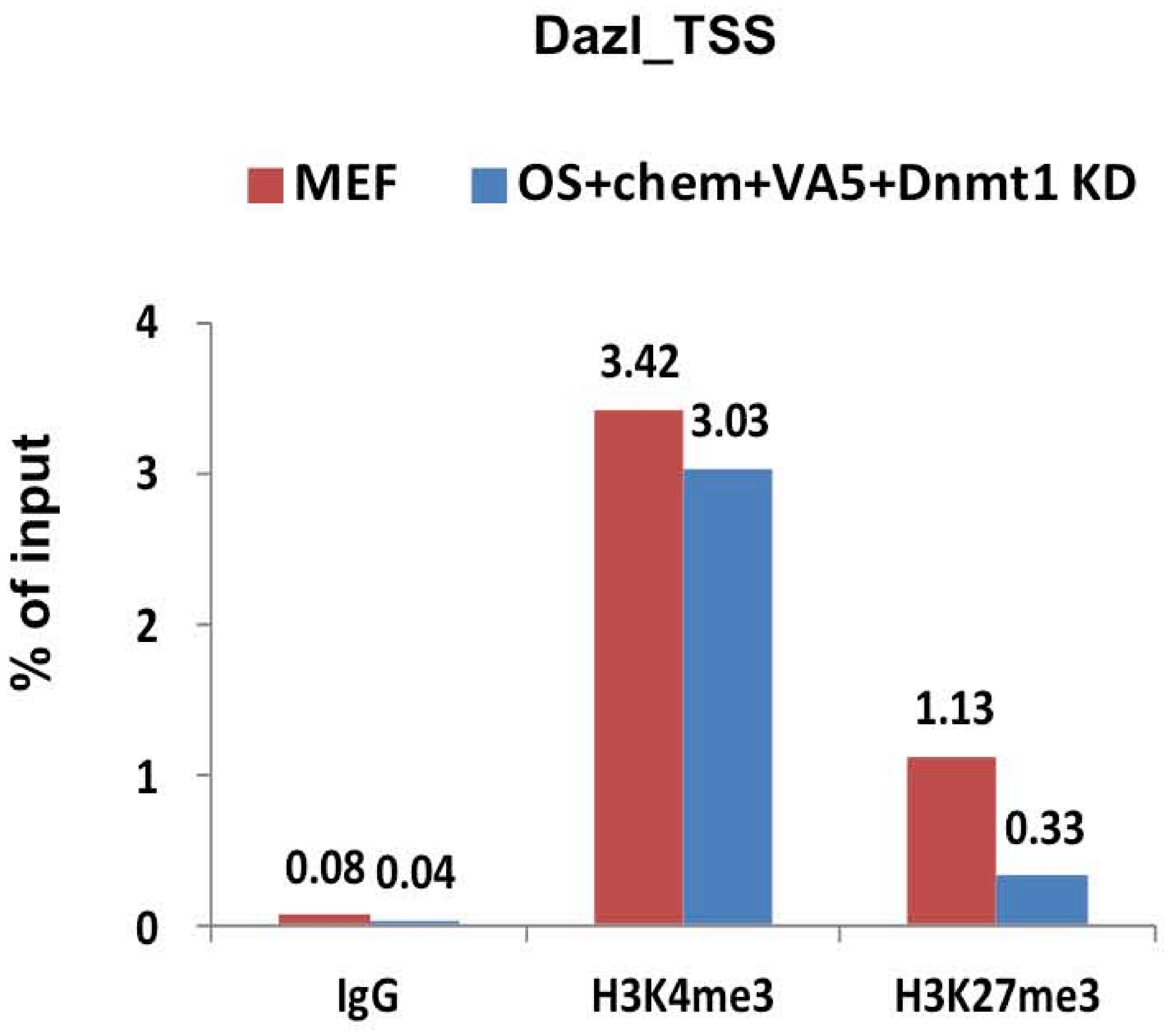

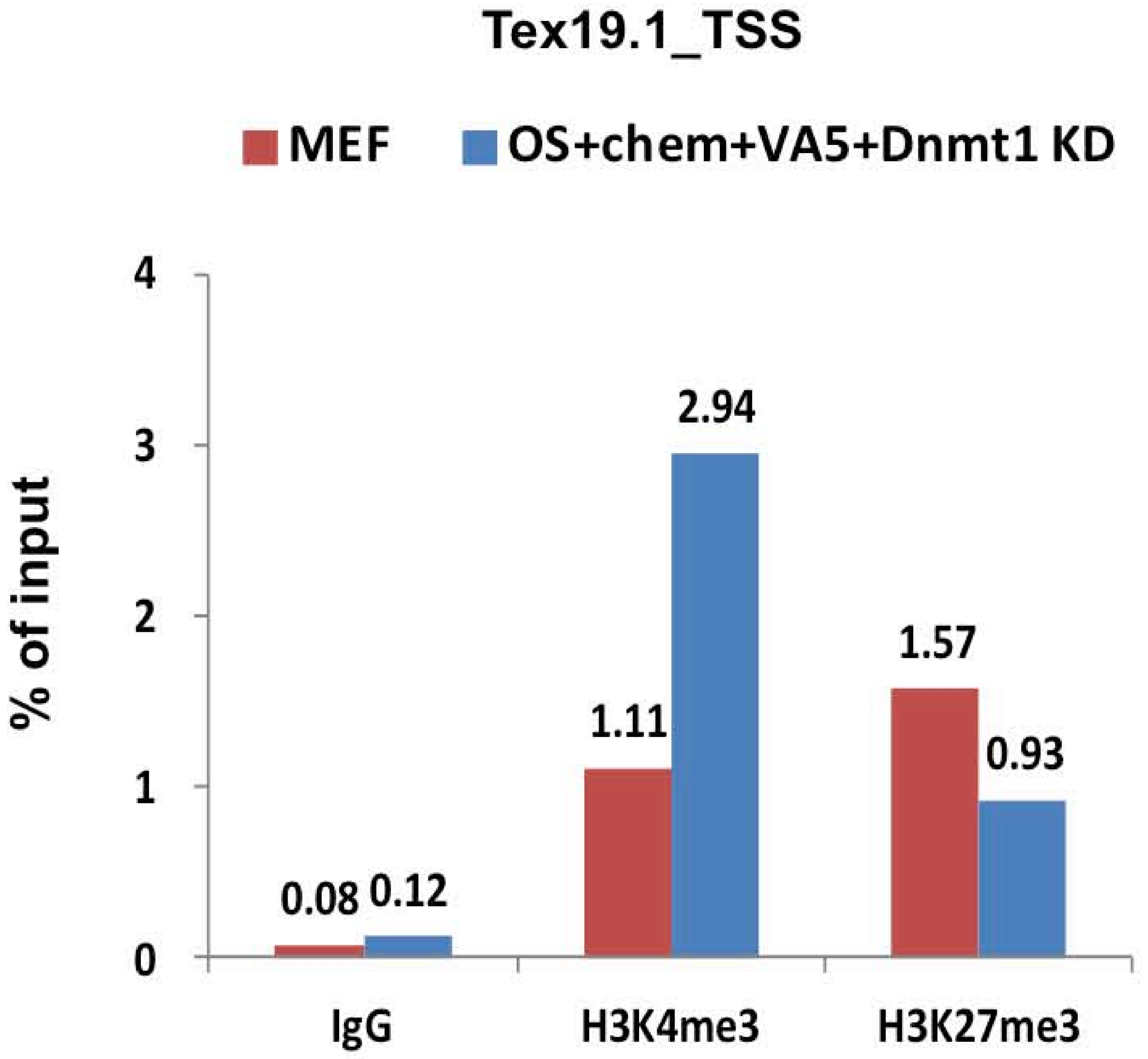

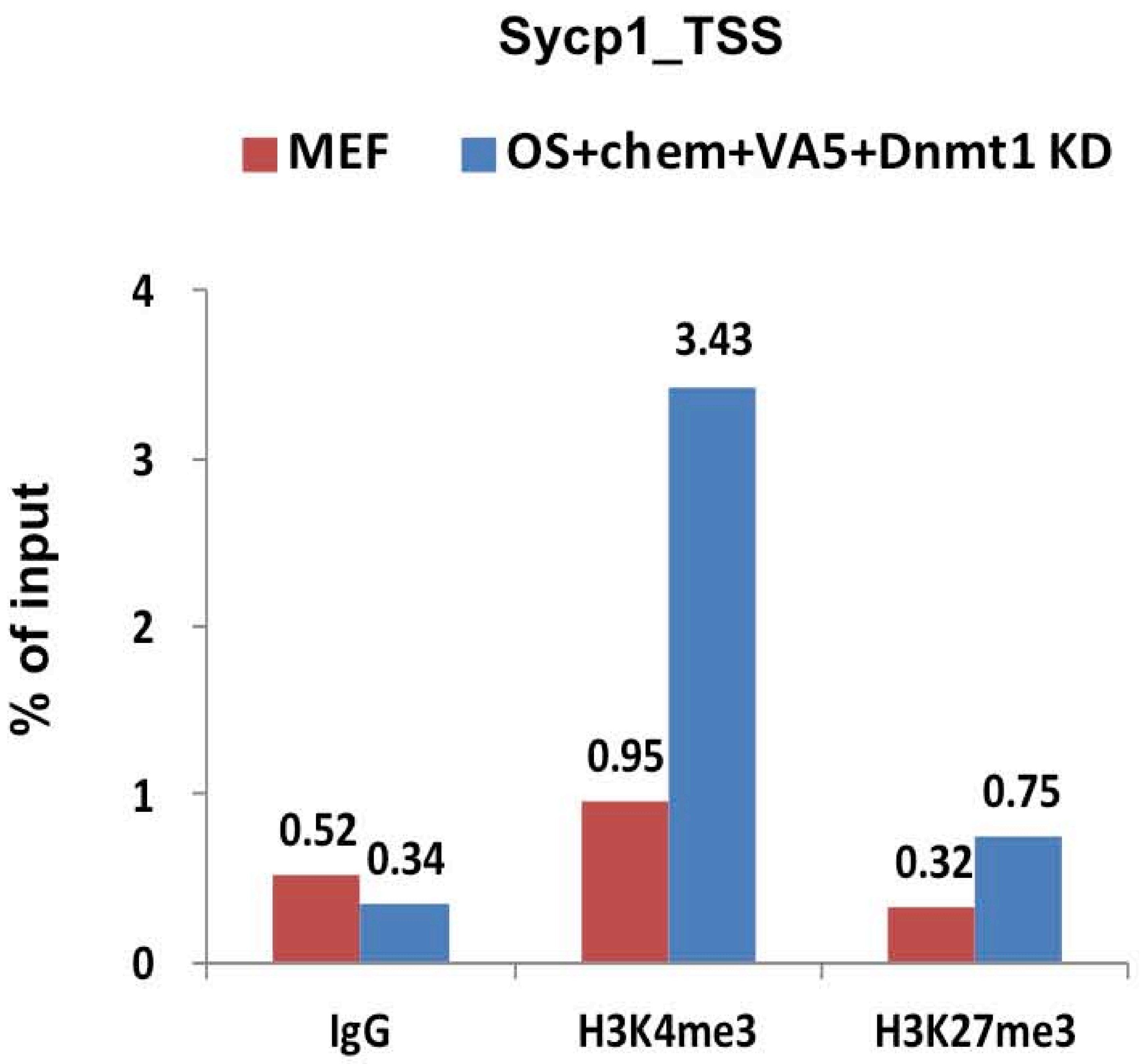

Supplementary Figure-S12 (Matsui)

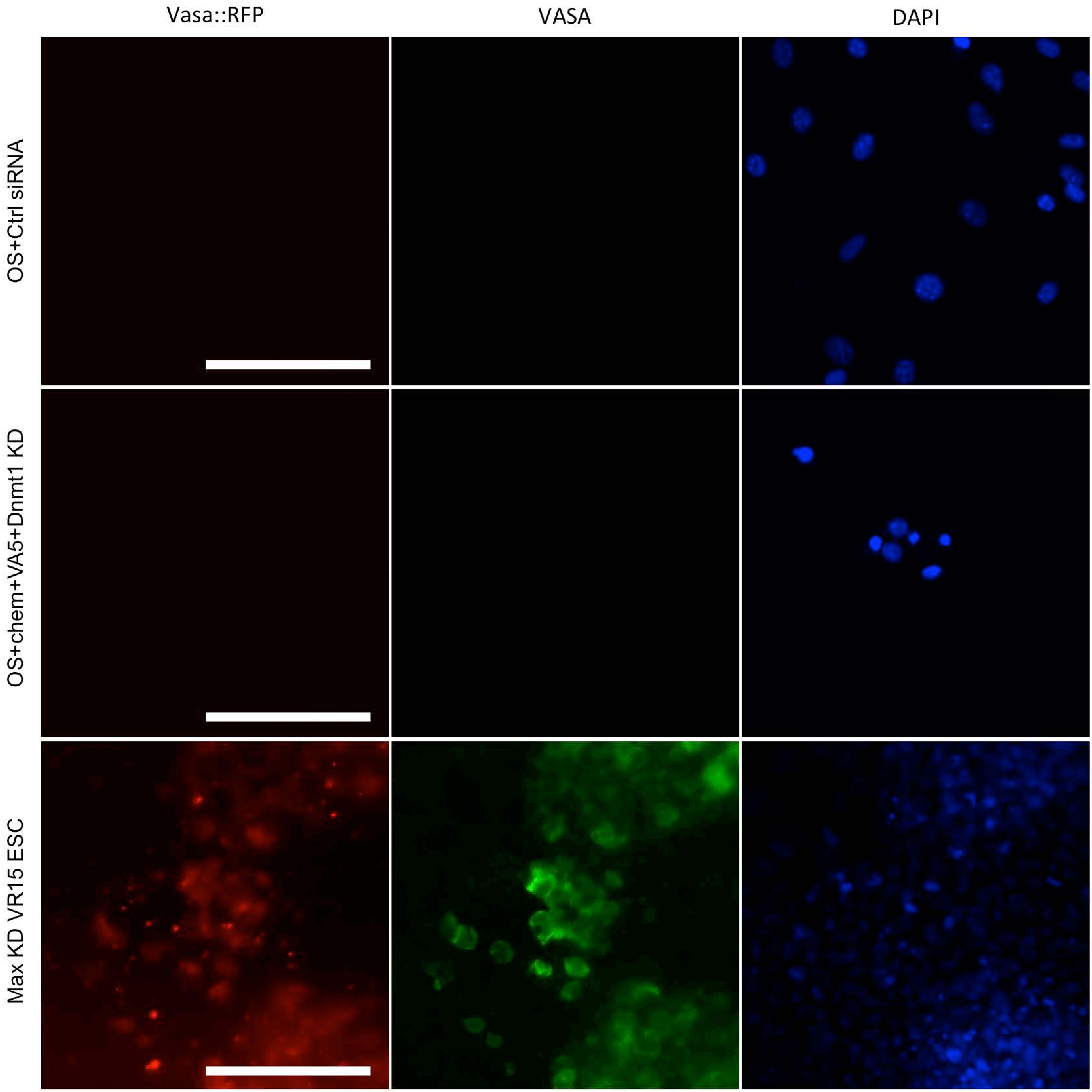

Supplementary Figure-S13 (Matsui)

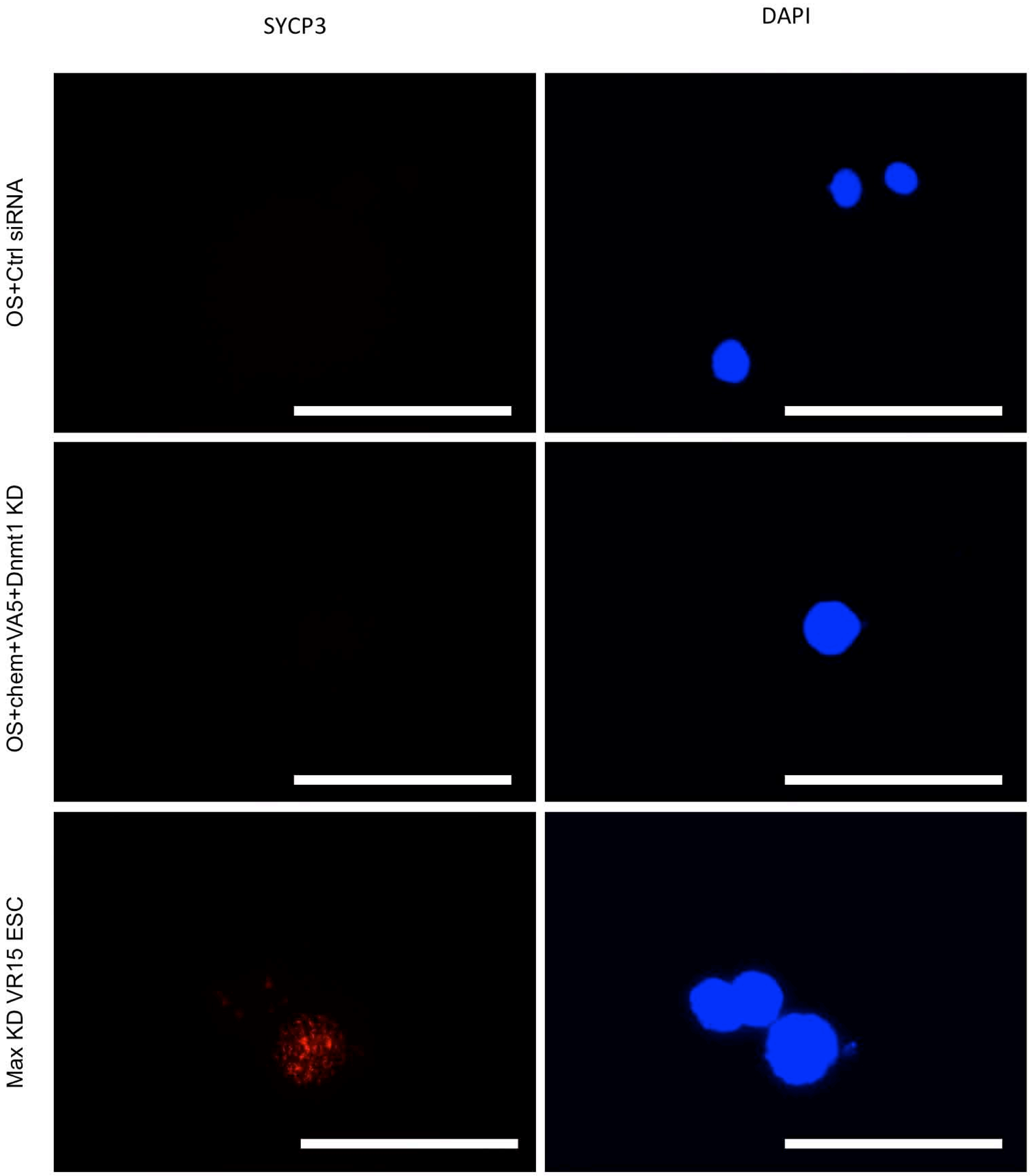
SYCP3
DAPI
OS+Ctrl siRNA
OS+chem+VA5+Dnmt1 KD
Max KD VR15 ESC

a

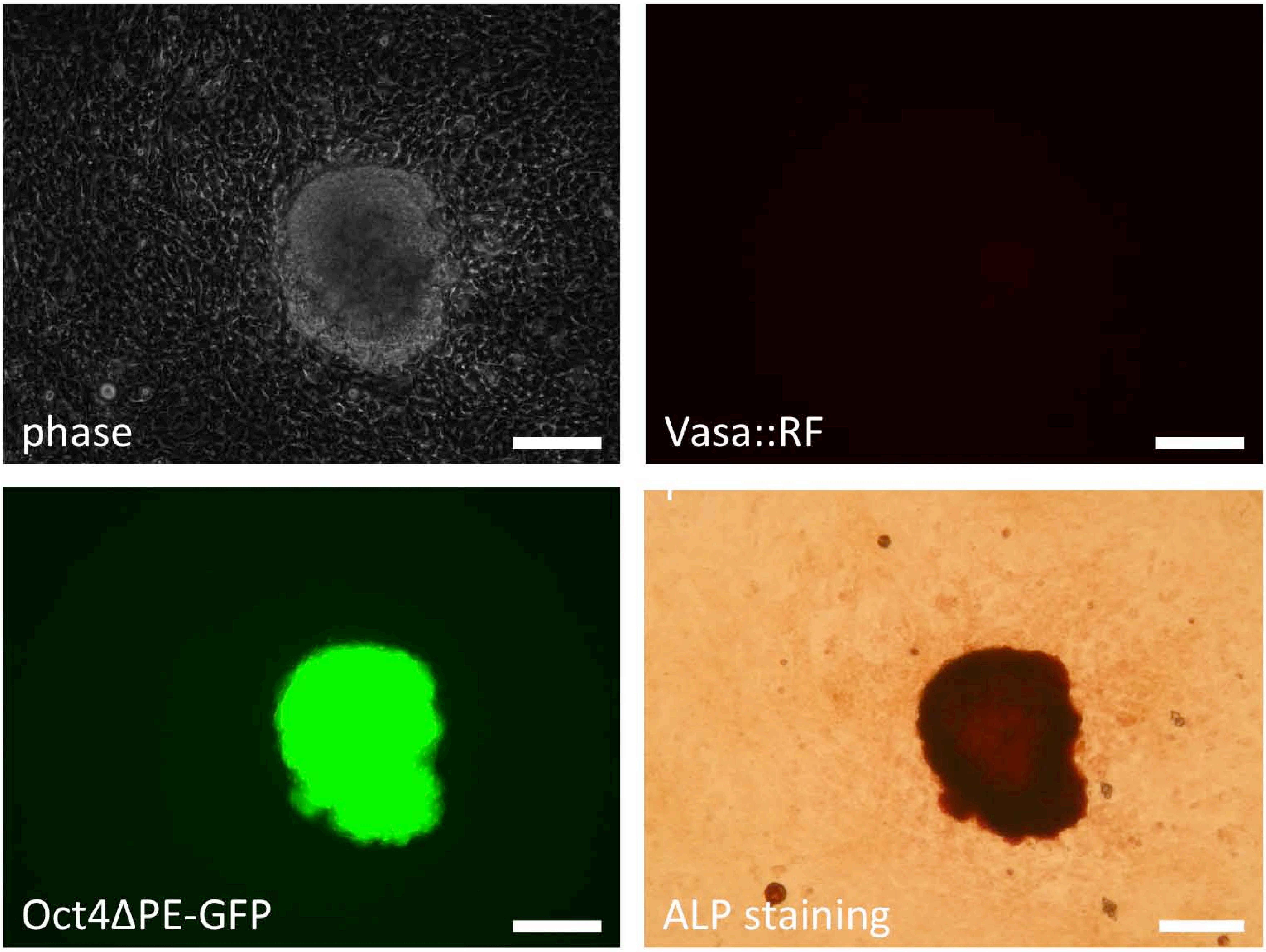
phase
Vasa::RF
Oct4ΔPE-GFP
ALP staining

b

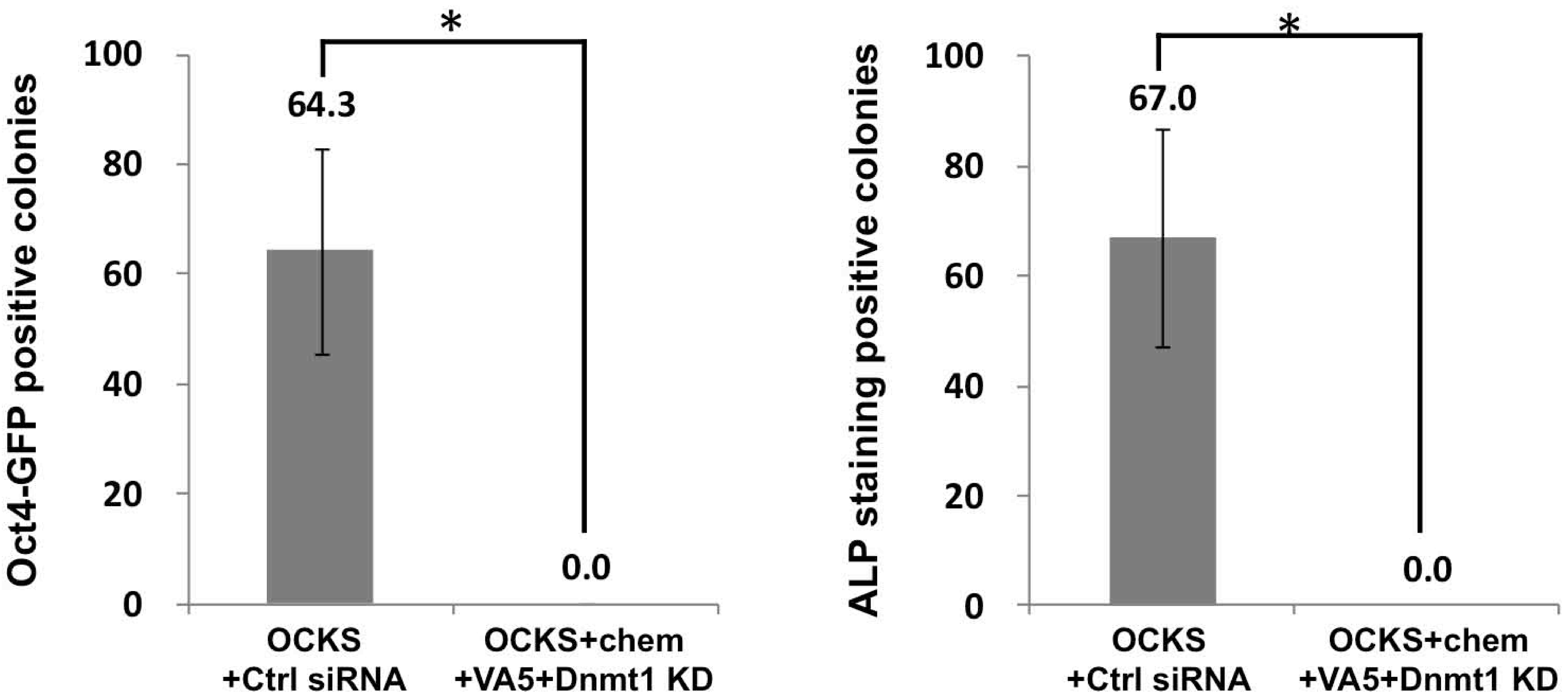
Oct4-GFP positive colonies
100
80
60
40
20
0
*
64.3
0.0
OCKS
+Ctrl siRNA
OCKS+chem
+VA5+Dnmt1 KD
ALP staining positive colonies
100
80
60
40
20
0
*
67.0
0.0
OCKS
+Ctrl siRNA
OCKS+chem
+VA5+Dnmt1 KD

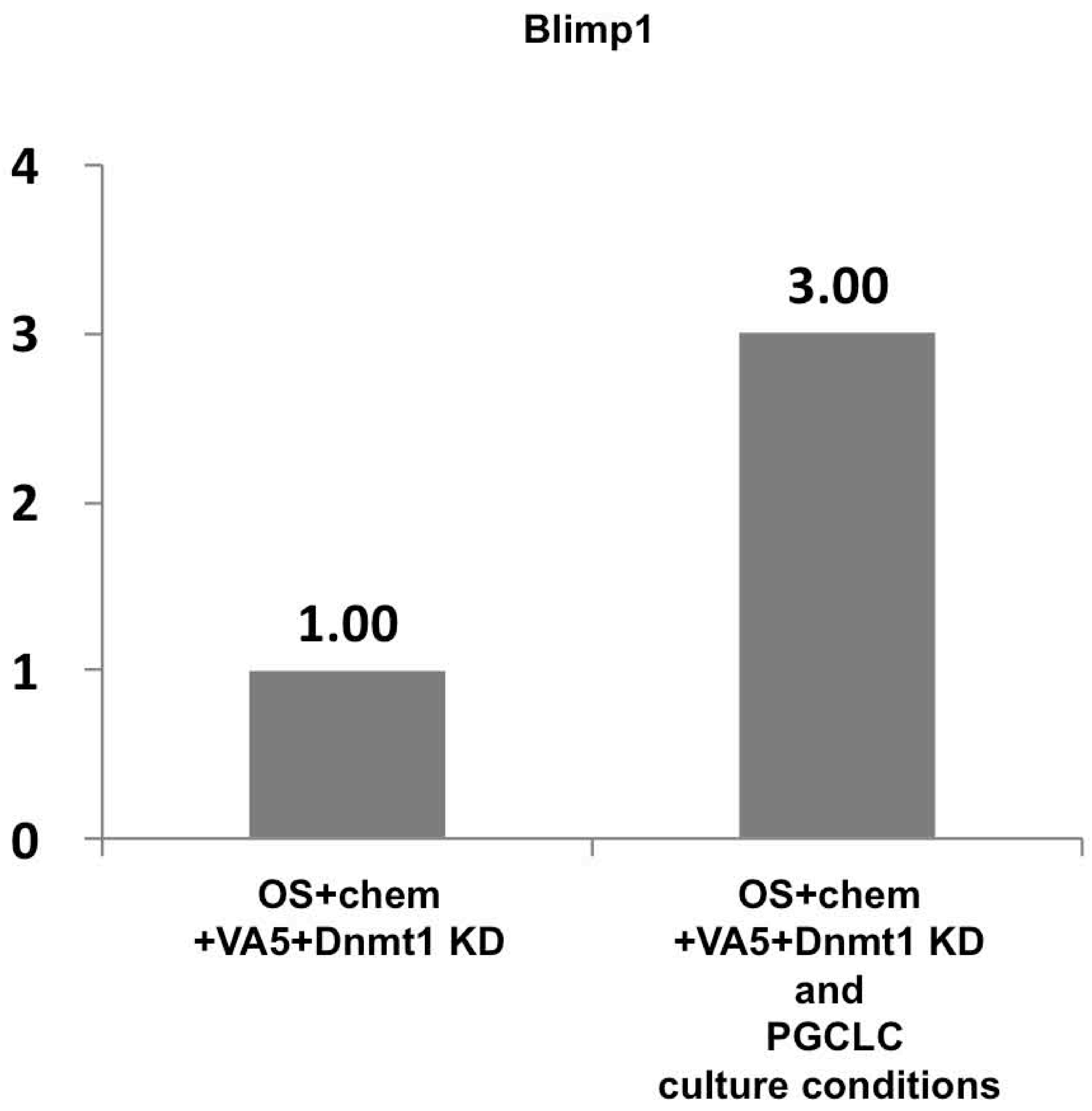

Supplementary Figure-S16 (Matsui)

Supplementary Table-S1 (Matsui)
Details of small-molecule compounds

Name	Source	solvent	Concentration (μM)
Valploic Acid : VPA	SIGMA (Cat. No. P4543-10G)	Water	0.25 mM
ALK5i (RepSox)	SIGMA (Cat. No. P0158-5MG)	DMSO	5 μM
Tranylcypromine	Enzo Life Sciences (Cat. No. BML-EI217-0001)	Water	2.5 μM
BIX-01294	STEMGENT (Cat. No. 04-0002)	DMSO	0.5 μM
3-Deazaneplanocin : DZNep	Selleck Chemicals (Cat. No. S7120)	Water	50 nM
5'-Azacytidine : Azacytidine	Active Motif (Cat. No. 14103)	Water	10 μM

Supplementary Table-S2 (Matsui)
Primers for real-time qPCR, bisulfite sequencing, ChIP-qPCR

Using	Target	Forward	Reverse
real-time qPCR	Ppia	GAGCTGTTTGCAGACAAAGTTC	CCCTGGCACATGAATCCTGG
	Arbp	AGATTCGGGATATGCTGTTGGC	TCGGGTCCTAGACCAGTGTTC
	Vasa	GGACGAGATTTGATGGCTTGTGC	AGCGACTGGCAGTTATTCCATCC
	Dazl	TGACGTGGATGTGCAGAAGATAG	CAAAGGACGTGGCTGCACATGA
	Stra8	CAGCTTAGAGGAGGTCAAGGAAG	AGCATCTGGTCCAACAGCCTCA
	Blimp1	TTCTCTTGGAAAAACGTGTGGG	GGAGCCGGAGCTAGACTTG
	Stella	CCAAGAGAAGGGTCCGCACTTT	GCAGAGACATCTGAATGGCTCAC
	Nanos3	GGGCTACACTTCTGTCTACTGC	TTCCTGCCACTTTTGGAACCT
	Hoxa1	AGCCACCAAGAAGCCTGTCGTT	TTGACCCACGTAGCCGTACTCT
	Hoxb1	GAATCGCCTTGCTCGTCAGAAC	CGTGGTGAAGTTTGTGCGGAGA
bisulphite sequencing	Dazl_outer	GTGGTTTGGTTATTGTTGTGTTTTT	TCCTACCACTCCCTAACCCC
	Dazl_inner	GGAAGAAAAAAATTAAGTTTTGATGGT	TCCTACCACTCCCTAACCCC
	Tex19.1_outer	TGTGTTTAGGTTGTAGTTGAGAATTGAG	ATAAACTCCAAAACCAAACTCTCCTC
	Tex19.1_inner	TGTGTTTAGGTTGTAGTTGAGAATTGAG	AACTCATTTACATATCTCCCATAAAATCC
	Sycp1_outer	ATGGGGTTTATGGTTGTAGTATAGGATTTA	ACTTCCCCTCAAAACCTCCTAAAA
	Sycp1_inner	TTATGGTTGTAGTATAGGATTTATGTTTGG	CCCTCAAAACCTCCTAAAAACC
ChIP-qPCR	Dazl_TSS	CTACGTGAGGTGGCTGCCTA	CCTATTGGCTGTAGCACGTCA
	Tex19.1_TSS	GGGACCTCATGGGAGATATGTA	CTCTCCTCGCTAAGCACTGAA
	Sycp1_TSS	TGGACCAACCGTTAAATTGAG	CCTTTATGAAGACGACATGGAAC

Supplementary Table-S3 (Matsui)
A list of meiosis-related genes commonly upregulated in MEFs cultured in OS+chem+VA5+Dnmt1 KD condition and in E13.5 male PGCs (related to Fig. 4d)

Probe Name	Fold change	GeneSymbol	GenbankAccession	GeneName
A_51_P245533	557.9	Tex19.1	NM_028602	testis expressed gene 19.1
A_52_P271166	137.5	Dazl	NM_010021	deleted in azoospermia-like
A_55_P2185143	45.6	Fkbp6	NM_001277891	FK506 binding protein 6
A_55_P2018403	33.0	Sycp1	NM_011516	synaptonemal complex protein 1
A_51_P444137	25.9	Syce1	NM_001143765	synaptonemal complex central element protein 1
A_52_P346225	23.7	Asz1	NM_023729	ankyrin repeat, SAM and basic leucine zipper domain containing 1
A_55_P1989369	18.4	Tdrd9	NM_029056	tudor domain containing 9
A_51_P116609	17.0	Tex12	NM_025687	testis expressed gene 12
A_55_P1974869	15.2	Hormad1	NM_001289537	HORMA domain containing 1
A_55_P1952385	14.0	Fkbp6	NM_001277891	FK506 binding protein 6
A_55_P2168168	13.3	Tex11	NM_031384	testis expressed gene 11
A_55_P2008417	12.9	Mnd1	NM_029797	meiotic nuclear divisions 1 homolog (S. cerevisiae)
A_66_P138584	12.8	Mnd1	NM_029797	meiotic nuclear divisions 1 homolog (S. cerevisiae)
A_51_P274465	12.5	Sycp3	Y08485	synaptonemal complex protein 3
A_55_P1973427	9.5	Tex14	NM_001199293	testis expressed gene 14
A_55_P1986208	8.9	Ccnb1ip1	NM_001111119	cyclin B1 interacting protein 1
A_51_P494751	8.0	Mael	NM_175296	maelstrom homolog (Drosophila)
A_51_P329818	7.7	Dpep3	NM_027960	dipeptidase 3
A_55_P2018407	7.4	Sycp3	NM_011517	synaptonemal complex protein 3
A_51_P497870	6.4	Hormad1	NM_026489	HORMA domain containing 1
A_55_P2093705	5.8	Meig1	NM_008579	meiosis expressed gene 1
A_51_P494822	5.7	Stag3	NM_016964	stromal antigen 3
A_51_P244303	5.6	Smc1b	NM_080470	structural maintenance of chromosomes 1B
A_55_P2064180	5.2	Hormad1	NM_001289534	HORMA domain containing 1
A_51_P242967	4.8	Piwil2	NM_021308	piwi-like RNA-mediated gene silencing 2
A_52_P70787	4.5	Brdt	NM_054054	bromodomain, testis-specific
A_51_P142421	4.0	Rspo1	NM_138683	R-spondin homolog (Xenopus laevis)
A_55_P2093704	4.0	Meig1	NM_008579	meiosis expressed gene 1
A_55_P2046753	3.0	Piwil4	NM_177905	piwi-like RNA-mediated gene silencing 4
A_51_P125679	2.2	Rec114	NM_028598	REC114 meiotic recombination protein
A_55_P2011121	2.1	Meiob	NM_029197	meiosis specific with OB domains

Supplementary Table-S4 (Matsui)
A list of meiosis-related genes commonly upregulated in MEFs cultured in OCKS+chem+VA5+Dnmt1 KD condition and in E13.5 male PGCs (related to Supplementary Fig. S8d)

Probe Name	Fold change	GeneSymbol	GenbankAccession	GeneName
A_51_P245533	797.3	Tex19.1	NM_028602	testis expressed gene 19.1
A_52_P271166	111.7	Dazl	NM_010021	deleted in azoospermia-like
A_55_P2185143	59.6	Fkbp6	NM_001277891	FK506 binding protein 6
A_55_P2018403	28.5	Sycp1	NM_011516	synaptonemal complex protein 1
A_51_P444137	28.4	Syce1	NM_001143765	synaptonemal complex central element protein 1
A_52_P346225	25.2	Asz1	NM_023729	ankyrin repeat, SAM and basic leucine zipper domain containing 1
A_55_P1989369	22.1	Tdrd9	NM_029056	tudor domain containing 9
A_55_P1952385	18.7	Fkbp6	NM_001277891	FK506 binding protein 6
A_55_P1974869	16.0	Hormad1	NM_001289537	HORMA domain containing 1
A_51_P116609	14.3	Tex12	NM_025687	testis expressed gene 12
A_55_P2168168	13.3	Tex11	NM_031384	testis expressed gene 11
A_55_P2008417	12.4	Mnd1	NM_029797	meiotic nuclear divisions 1 homolog (S. cerevisiae)
A_51_P274465	12.3	Sycp3	Y08485	synaptonemal complex protein 3
A_66_P138584	12.2	Mnd1	NM_029797	meiotic nuclear divisions 1 homolog (S. cerevisiae)
A_55_P2018407	9.3	Sycp3	NM_011517	synaptonemal complex protein 3
A_55_P1973427	8.8	Tex14	NM_001199293	testis expressed gene 14
A_55_P1986208	8.5	Ccnb1ip1	NM_001111119	cyclin B1 interacting protein 1
A_51_P494822	7.8	Stag3	NM_016964	stromal antigen 3
A_51_P329818	7.5	Dpep3	NM_027960	dipeptidase 3
A_55_P2093705	6.4	Meig1	NM_008579	meiosis expressed gene 1
A_51_P494751	6.1	Mael	NM_175296	maelstrom homolog (Drosophila)
A_51_P244303	5.6	Smc1b	NM_080470	structural maintenance of chromosomes 1B
A_51_P242967	5.3	Piwil2	NM_021308	piwi-like RNA-mediated gene silencing 2
A_55_P2064180	4.9	Hormad1	NM_001289534	HORMA domain containing 1
A_51_P497870	4.9	Hormad1	NM_026489	HORMA domain containing 1
A_55_P2093704	4.4	Meig1	NM_008579	meiosis expressed gene 1
A_52_P70787	3.8	Brdt	NM_054054	bromodomain, testis-specific
A_55_P2046753	2.5	Piwil4	NM_177905	piwi-like RNA-mediated gene silencing 4
A_66_P107007	2.5	Msh4	NM_031870	mutS homolog 4 (E. coli)
A_55_P2208041	2.1	Stra8	NM_009292	stimulated by retinoic acid gene 8
A_51_P125679	2.1	Rec114	NM_028598	REC114 meiotic recombination protein
